# Distribution of glutathione peroxidase-1 immunoreactive cells in pancreatic islets from type 1 diabetic donors and non-diabetic donors with and without islet cell autoantibodies is variable and independent of disease

**DOI:** 10.1007/s00441-025-03955-5

**Published:** 2025-03-10

**Authors:** Kaaj Pala, Kevin Xueying Sun, Lars Krogvold, Knut Dahl-Jørgensen, Shiva Reddy

**Affiliations:** 1 Department of Molecular Medicine and Pathology, Faculty of Medical and Health Sciences, University of Auckland, Auckland, 1023 New Zealand; 2 Division of Paediatric and Adolescent Medicine, Oslo University Hospital, Oslo, Norway; 3 Faculty of Dentistry, University of Oslo, Oslo, Norway; 4 Faculty of Medicine, University of Oslo, Oslo, Norway; 5 Oslo University Hospital, Oslo, Norway

**Keywords:** Type 1 diabetes, Glutathione peroxidase-1, Reactive oxygen species, Beta cell damage, Immunohistochemistry

## Abstract

During type 1 diabetes (T1D), oxidative stress in beta cells may cause early beta cell dysfunction and initiate autoimmunity. Mouse islets express lower levels of reactive oxygen species (ROS) clearing enzymes, such as glutathione peroxidase (GPX), superoxide dismutase (SOD) and catalase than several other tissues. It remains unclear if human beta cells show a similar deficiency during T1D or exhibit a higher degree of intrinsic resistance to oxidative stress. We compared islet cell distributions and determined graded intensities of glutathione peroxidase1 (GPX1), a key enzymatic mediator involved in detoxifying hydrogen peroxide, by applying combined immunohistochemistry for GPX1, insulin and glucagon, in pancreatic sections from new-onset T1D (group 1), non-diabetic autoantibody-negative (group 2), non-diabetic autoantibody-positive (group 3) and long-term diabetic (group 4) donors. Islets from all study groups demonstrated either uniform but graded staining intensities for GPX1 in almost all islet cells or strong staining in selective islet cells with weaker intensities in the remaining cells. GPX1 was present in selective glucagon cells and insulin cells, including in cells negative for both hormones, with stronger intensities in a higher percentage of glucagon than insulin cells. It was absent in a higher percentage of beta cells than glucagon cells independent of disease or autoantibody positivity. We conclude that a proportion of human beta cells and glucagon cells express GPX1 but show heterogeneity in its distribution and intensities, independent of disease or autoantibody status. Our studies highlight important differences in the expression of GPX1 in islet cell-types between mice and humans.

**Supplementary Information:**

The online version contains supplementary material available at 10.1007/s00441-025-03955-5.

## Introduction

During type 1 diabetes (T1D), beta cells in pancreatic islets are selectively and progressively destroyed by immune-mediated processes, culminating in an abrupt clinical onset (Atkinson et al. 2011, 2014). Its diagnosis is preceded by an asymptomatic protracted phase lasting several months to years when subjects manifest variable degrees of beta cell dysfunction (Atkinson and Mirmira 2023, Evans-Molina et al. 2018). Following onset, daily administration of insulin is mandatory to suppress hyperglycaemia. The immune-mediated destructive process is beta cell specific since the closely located non-beta endocrine cells within the islet escape destruction, although glucagon cells display functional impairment (Atkinson and Mirmira 2023, Doliba et al. 2022, Yosten 2018). The exquisite immune specificity for beta cells suggests that such cells may already harbour molecular signatures of vulnerability, which may act not only as initiators or conduits for the early immune response but also as amplifiers of ongoing beta cell-directed immune destruction (Kracht et al. 2017; Mallone and Eizirik 2020; Mallone et al. 2022; Peters et al. 2019; Roep et al. 2021; Thompson et al. 2023). However, a subset of beta cells in some subjects remain for many years after diagnosis (Keenan et al. 2010; Reddy et al. 2021).

Although low levels of reactive oxygen species (ROS) mediate critical physiological signaling pathways, accumulation of raised levels, if not cleared rapidly, can lead to major deleterious consequences affecting several organs, such as the heart, kidney, eye and brain, culminating in a variety of human disorders (Forman and Zhang 2021). Elevated ROS injure cells by promoting degradation of cellular proteins, lipids and DNA, subvert beta cell biosynthetic capacity and cause endoplasmic reticulum (ER) stress or entwine with it (Hasnain et al. 2016). In addition, they affect several adverse cell signaling pathways involving mammalian target of rapamycin complex 1 (mTORC1) and c-jun N-terminal kinase (JNK) and also invoke inflammation by activating nuclear factor-kB (nF-κB) (Evans-Molina et al. 2018, Hasnain et al. 2016, Kulkarni et al. 2023).

Beta cells exhibit inherent sustained high respiratory, metabolic, secretory and protein synthetic rates (Evans-Molina et al. 2018). High respiratory activity results in raised levels of ROS originating from the electron transport chain. However, little is known about how human beta cells efficiently eliminate excess levels of ROS. During T1D, early environmental agents, such as certain viruses and chemicals, may also provoke deleterious events in beta cells, leading to oxidative, nitrosative and endoplasmic reticulum (ER) stress (Amirruddin et al. 2021; Craig et al. 2019; Hasnain et al. 2016; Hober and Sauter 2010; Leenders et al. 2021; Mallone and Eizirik 2020; Roep et al. 2021). Such stressors result in the formation of misfolded proteins and neoantigens, which trigger a clinically silent phase of beta cell dysfunction and steer self-directed immune reactivity before clinical diagnosis (Atkinson et al. 2011; Sims et al. 2019, 2020). For example, recent studies have shown that ER stress within beta cells leads to translational errors in the reading frame of human insulin mRNA, resulting in the generation of defective polypeptides and the formation of neo-epitopes, capable of recognition by circulating T cell populations which have escaped prior thymic deletion (Kracht et al. 2017; Roep et al. 2021). In addition, neo-epitope formation from degraded citrullinated proteins, such as glucose-regulated protein 78 following exposure of human islets to inflammatory molecules, can lead to immune responses against beta cells (Buitinga et al. 2018).

High levels of ROS, such as superoxide anions (O_2_^◦ −^) and hydrogen peroxide (H_2_O_2_), originate from the electron transport chain and, to a lesser extent, following catalysis by cytosolic and plasma membrane oxidoreductases (Nolfi-Donegan et al. 2020). Excess levels of superoxide can dismutate either spontaneously to H_2_O_2_ or following catalysis by superoxide dismutase (SOD). Hydrogen peroxide is converted to water and oxygen by glutathione peroxidases, catalase and peroxiredoxins (Bast et al. 2002; Handy and Loscalzo 2022; Stancill et al. 2019; Stancill and Corbett 2021; Wolf et al. 2010). Mouse islets express lower levels of SOD, glutathione peroxidase (GPX) and catalase at the mRNA and protein level than other tissues such as the liver and kidneys (Grankvist et al. 1981; Lenzen 2008; Tiedge et al. 1997, 1999). During the prodromal phase of T1D and preceding early insulitis, islet cell autoantibodies against several antigens such as insulin, glutamic acid decarboxylase and zinc transporter-8 are detectable in sera of most subjects (Bogun et al. 2020; Campbell-Thompson et al. 2016; Krischer et al. 2022). Whether excess uncleared ROS originating from beta cell mitochondrial respiration contributes to the ontogeny of islet cell autoantibodies and insulitis remains unclear.

Glutathione peroxidase (GPX) is present in six isoforms. Isoforms 1 to 4 are homotetramers containing selenium bound to cysteine in their active site and are referred as selenoproteins (Handy and Loscalzo 2022). They play a key role in detoxifying H_2_O_2_ and lipid peroxides in multiple tissues (Robertson and Harmon 2007; Tanaka et al. 1999). Glutathione peroxidase catalysis occurs in the presence of reduced glutathione, a tripeptide, in a paired reaction involving glutathione reductase, which regenerates reduced glutathione from oxidized glutathione (Handy and Loscalzo 2022). However, whether the previously reported lower level of glutathione peroxidase-1 (GPX1) demonstrated in mouse and human islets by Western blotting and enzyme assays is also an intrinsic feature of human beta cells in situ is unclear (Grankvist et al. 1981; Tonooka et al. 2007).

Little is known about the cellular expression of GPX1 in human pancreatic islets in donors with increasing duration of T1D and if the expression patterns differ in non-diabetic cases with and without islet cell autoantibodies. In a brief study, immunohistochemical analysis of pancreatic sections from deceased non-diabetic human donors showed expression of GPX1 and catalase predominantly in selective glucagon cells and some beta cells, with an absence in the remaining islet cell-types (Miki et al. 2018). However, this study did not fully analyze the possible changing expression pattern of GPX1 in glucagon and beta cells from donors with variable duration of T1D, including new-onset cases and in autoantibody-positive and autoantibody-negative non-diabetic donors. Thus, there is an urgent need to investigate and compare the expression of GPX1 in persisting beta cells and other islet cell-types at and after diagnosis of T1D with non-diabetic cases with and without islet cell autoantibodies.

The recent availability of rare pancreatic samples from cases with variable but precisely defined duration of T1D and from non-diabetic donors with and without serum islet cell autoantibodies has provided us with an opportunity and justification to undertake detailed studies on the expression of GPX1 in islets. In the present study, we have, therefore, utilized these unique samples and applied an optimized triple-label immunohistochemical protocol for comparing the localization of GPX1 in beta and alpha cells and for estimating its level and relative expression in the two cell-types in high-quality human pancreatic sections from diabetic and non-diabetic deceased donors, supplied by the Network for Pancreatic Organ Donors with Diabetes (nPOD) and the Exeter Archival Diabetes Biobank (EADB) (Morgan and Richardson 2018; Pugliese et al. 2014). We have also analyzed sections of well-preserved surgically retrieved rare pancreatic biopsies from four living volunteers with recent-onset T1D, supplied by the Diabetes Virus Detection (DiViD) Study (Krogvold et al. 2014).

## Methods

### Subjects and pancreatic sections

Paraffin-embedded pancreatic sections (5 µm) from formalin-fixed biopsy specimens surgically excised from the pancreatic tail region of 4 living individuals with newly diagnosed type 1 diabetes from DiViD, 22 formalin-fixed cadaveric pancreases also from the pancreatic tail supplied by nPOD and 1 pancreas at post-mortem from EADB, UK, were analyzed (Krogvold et al. 2014; Morgan and Richardson 2018; Pugliese et al. 2014). In addition, sections prepared from formalin-fixed liver (University of Harbin, Harbin, China), breast (Auckland Tissue Biobank, University of Auckland) and lung (Department of Anatomy and Medical Imaging, University of Auckland) carcinoma were tested as positive controls for GPX1 immunostaining. Pancreatic sections from human cases were triple-immunostained for GPX1, insulin and glucagon and analyzed. Please refer to “Acknowledgements” for ethical approval.

The main study groups are summarized in Table 1, and donor demographics are detailed in the electronic supplementary material (ESM Table 1).

**Table 1:** Summary of main study groups, including sex and age distribution

Study group	Sex (no. female/no. male)	Mean age (years)	Median age (years)	Age range (years)
Group 1, new-onset diabetes	**2/2**	**28.5**	**28**	**24–31**
Group 2, non-diabetic AAb-negative	**4/5**	**26.97**	**25.1**	**19–44**
Group 3, non-diabetic AAb-positive	**2/4**	**28.66**	**27**	**17.65–40.3**
Group 4, long-term diabetes	**4/4**	**28.08**	**28.8**	**22–44**

AAb, autoantibody

### Triple-label immunohistochemical protocol

We employed a commercially available affinity-purified rabbit polyclonal antibody monospecific for human GPX1 (Abcam ab22604, supplied as 1 mg/ml IgG). The suppliers have validated this antibody as suitable for immunohistochemistry and Western blotting (refer below for further details on immunohistochemical protocols and ESM Table 2). It has also been validated and employed by previous investigators for immunohistochemical localization of GPX1 in formalin-fixed human lung sections and Bouin’s fixed human pancreatic sections (Basnet et al. 2019, Miki et al. 2018). Anti-GPX1 was titrated to a working dilution of 1:120 (final concentration, 8.33 µg/ml IgG) before its application to sections from formalin-fixed human pancreas. As negative controls, anti-GPX1 was replaced with diluent or normal rabbit IgG in the immunohistochemical procedure. In initial studies, immunohistochemical staining for GPX1 in pancreatic sections with species-specific secondary antibodies linked to anti-rabbit IgG-horseradish peroxidase polymer and subsequent reaction with diaminobenzidine hydrochloride (adopted in this study) was compared with donkey anti-rabbit IgG-Alexa 568 as secondary antibody. The two protocols yielded concordant cellular staining (see below under “Methods”). In addition, we stained pancreatic sections with a mixture of rabbit anti-glucagon (same immunizing species as GPX1 antibody), guinea pig anti-insulin and mouse anti-CD45. Immunohistochemical specificity of anti-GPX1 was also evaluated in formalin-fixed paraffin sections from liver, breast and lung cancer, known to express GPX1 (Basnet et al. 2019, Woolston et al. 2011). Please see ESM Fig. 1 and ESM Fig. 2 under “Results”.

The immunohistochemical protocol for GPX1 applied in this study was slightly modified from our previous procedures for interleukin-1β and inducible nitric oxide synthase (Reddy et al. 2018; Reddy et al. 2021). Relevant immunohistochemical steps, reagents and incubation times are summarized in ESM Table 2. Thus, sections were deparaffinized, rehydrated and subjected to antigen retrieval with citrate buffer, pH 6, containing 0.05% (vol/vol) Tween-20 (Sigma-Aldrich, Darmstadt, Germany), cooled in distilled water and permeabilized in 0.4% (vol/vol) Triton-X-100 in phosphate-buffered saline (PBS), pH 7.4, on ice for 30 min. After washing sections in PBS for 10 min, they were exposed to 3% (vol/vol) H_2_O_2_ in PBS for 15 min. Following two consecutive 5-min washing steps in PBS, sections were blocked with 10% (vol./vol.) normal goat serum constituted in low protein blocking solution (eBioscience, San Diego, CA, USA) for 1 h at 37 °C, followed by incubation with rabbit anti-GPX1 (1:120, diluted in antibody diluent from Cell Signaling Technology, Danvers, MA, USA) for 16–18 h at 4 °C. After a wash step, sections were incubated with anti-rabbit IgG horseradish peroxidase polymer (Cell Signaling Technology), diluted 1:1 with 0.1% vol/vol Tween-20 in PBS (wash buffer). After further washing, sections were reacted with 3,3′-diaminobenzidine (DAB) substrate (freshly prepared in accordance with the manufacturer’s instructions; catalogue number: 8059; Cell Signaling Technology) for 5–6 min. After monitoring peroxidase-catalyzed DAB-product by bright field microscopy, the reaction was terminated in excess distilled water. Sections were then briefly immersed in wash buffer and incubated with a mixture of guinea pig anti-insulin and mouse anti-glucagon (both at 1:600 dilution in antibody diluent from Cell Signaling) and incubated for 90 min at 37 °C. Sections were washed in wash buffer and reacted with a mixture of highly cross-absorbed species-specific donkey anti-guinea pig IgG-Alexa 488 (1:300) and donkey anti-mouse IgG-Alexa 568 (1:600), diluted in 0.2% vol/vol Tween-20 in PBS for 1 h at 37 °C. Following washing, sections were mounted with CitiFluor mountant for microscopic analysis.

### Image acquisition

Sections were examined with a Nikon Eclipse-epifluorescence-bright field microscope, and digital images were recorded and saved as Tiffs. Groups 2 and 3 were blinded to the investigator, but not groups 1 and 4, where many islets from the two latter cases were either insulin-negative or showed reduced number of residual beta cells. Such distinct pathologies displayed by all cases from the two diabetic groups did not permit sample blinding. However, case characteristics such as duration of T1D and clinical information were blinded to the investigator. From each section, all islets with approximately ≥ 20 endocrine cells were imaged for the presence of GPX1 by bright field microscopy. Uniform gain and exposure settings in the Nikon NIS Imaging Software with identical objective lens magnification of × 40 with a numerical aperture of 0.75 were applied to all sections to ensure consistency in image intensities and magnification. Following image acquisition by bright field microscopy, the same islets were co-imaged for insulin and glucagon staining by fluorescence microscopy equipped with appropriate narrow-band filters. Each specific image set from multiple acquisitions (GPX1, ± insulin and glucagon) was merged using Adobe Photoshop CS6 (Adobe Systems, San Jose, CA, USA). For image display in combination with insulin and glucagon staining by fluorescence, GPX-1 immunostained cells (initially brown) were converted to a blue fluorescent pseudocolour for photographic display.

### Image analysis

Insulin-positive and insulin-negative islets in all donors were enumerated.

**Fig. 1:**
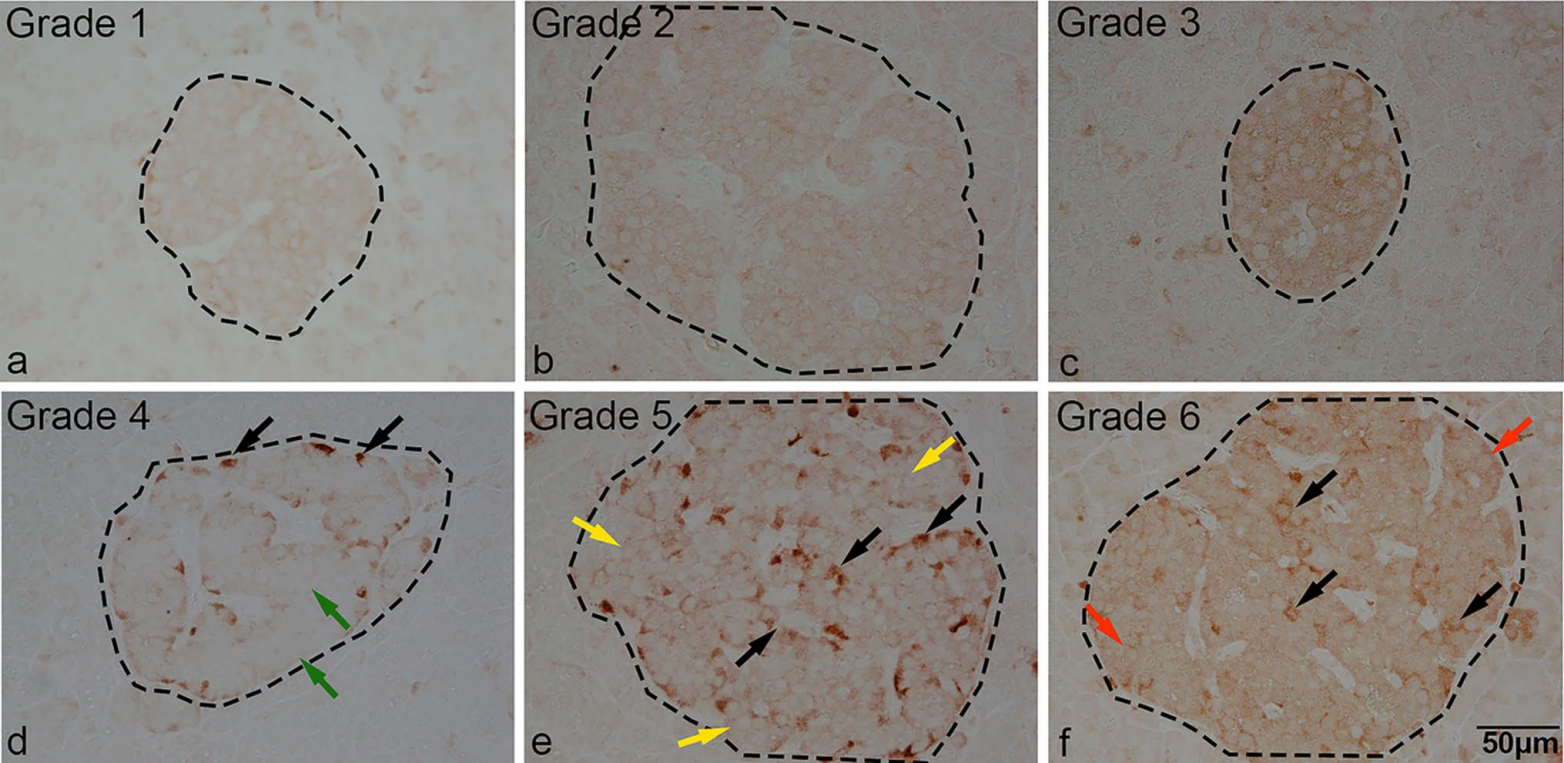
Grading system employed for estimating overall GPX1 staining intensities in various islets. The overall staining intensities in islet cells were graded on a score of 1–6, following a display of microscopic digital images of islets in Photoshop. Intensity scores in islets and the pattern of GPX1 staining were assigned as follows: **a** 1 = very weak staining of most islet cells; **b** 2 = moderate staining of most islet cells; **c** 3 = strong staining of most islet cells; **d** 4 = strong staining of a minority of islet cells (black arrows) with the majority showing negative staining in most islet cells (green arrows); **e** 5 = strong staining of a minority of islet cells (black arrows), with a majority showing weak staining in most islet cells (yellow arrows); **f** 6 = strong staining of a minority of islet cells (black arrows) with the majority of cells showing moderate staining in most islet cells (red arrows). Islet boundaries are indicated by black dashes. Scale bar in (**f**), 50 µm, applies to all micrographs. Grades 1–3 and 4–6 were assigned Pattern 1 and Pattern 2, respectively

Following triple staining (GPX1 ± insulin + glucagon), the overall cellular intensity for GPX1 (brown) and its distribution pattern in all islets with more than 20 estimated cells were graded visually following display of acquired images by Photoshop with sufficient magnification for ease of determining the distribution of cells as follows. Islets with very weak staining in almost all islet cells were assigned grade 1, those with overall weak staining as grade 2 and those with moderate to strong staining in most islet cells were assigned grade 3 (Pattern 1). In addition, islets with strong GPX1 staining in only a few selective islet cells with negative staining in a majority of islet cells were assigned grade 4, while those with strong staining in a minority of islet cells accompanied by weak staining in a majority of islet cells were assigned grade 5. Islets showing strong staining in a minority of cells coupled with moderate staining in most islet cells were assigned “grade 6”. Grades 4–6 were defined as “Pattern 2”. The grading system for the two patterns was based on a visual immunostained guide shown in Fig. 1.

**Fig. 2:**
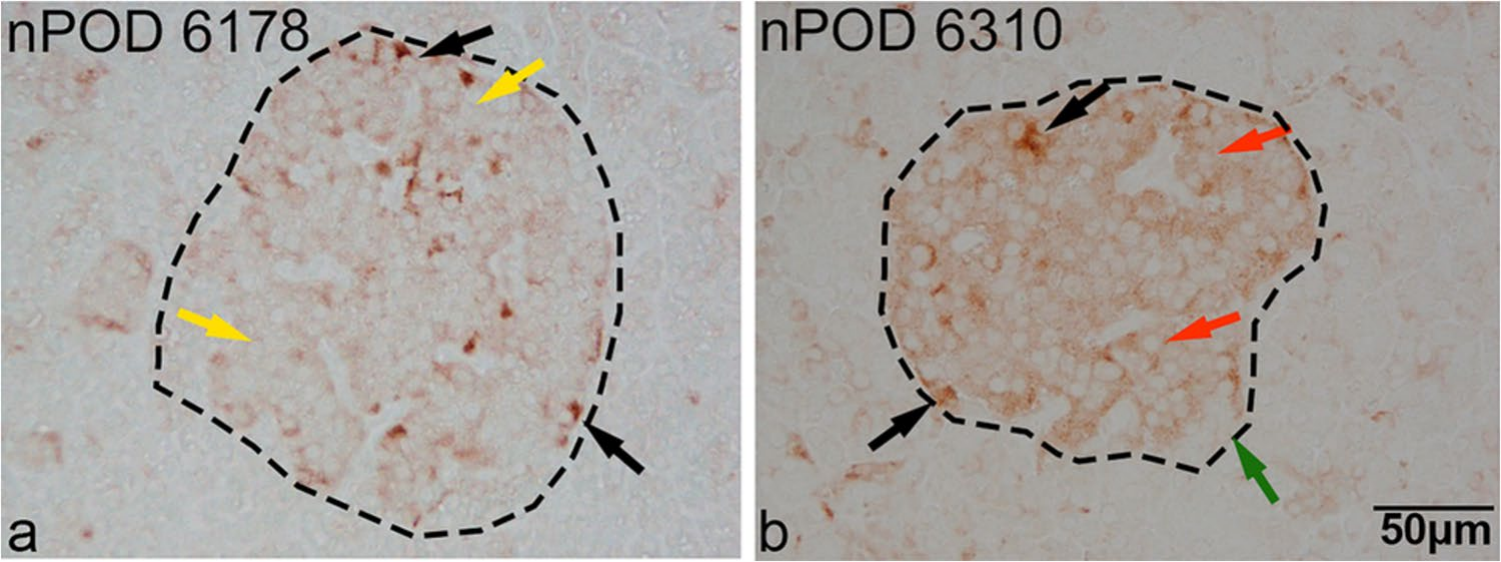
Images of 2 islet sections employed as a visual guide for grading differing cellular intensities of GPX1 staining in insulin and glucagon cells. Images of 10 randomly selected islets from each case triple-stained for GPX1, insulin and glucagon were merged in Photoshop and graded for GPX1 intensities in insulin and glucagon cells separately on a scale of negative (green arrows), weak (yellow arrows), moderate (red arrows) and strong (black arrows). Islet boundaries are indicated by black dashes. Scale bar in (**b**), 50 µm, applies to all micrographs

We also graded the intensities of GPX1 staining in each insulin and glucagon cell located in 10 randomly selected islets per case by viewing, magnifying and merging image sets in Photoshop to ascertain the precise identity of the two cell types, GPX-1 positivity, grading and enumeration. Staining for GPX1 in each insulin and glucagon cell from 10 islets per case was scored as either negative, weak, moderate or strong in accordance with a visual immunostained template guide (Fig. 2). In insulin-negative islets from diabetic cases, the various intensity grades for GPX1 were derived from glucagon cells.

### Data analysis

Results were tabulated and represented as bar graphs for each donor and analyzed for the following:

- Number of islets imaged per section
- Number of islets per case positive and negative for insulin
- Percentage of all islets, including insulin-positive and negative islets from each donor with GPX-1 positive cells and their intensities, graded on a scale from 1 to 3 (Pattern 1; Fig. 1) and 4 to 6 (Pattern 2; Fig. 1)
- Percentage of insulin and glucagon cells in 10 randomly selected islets from each case with various intensities of GPX-1 staining, graded on a scale of negative, weak, moderate and strong (indicated in Fig. 2)
- Total percentage of insulin and glucagon cells per study group with various grades of GPX1 staining as specified under (d). For each group, the total number of GPX1 cells corresponding to either insulin or glucagon cells was calculated separately for the two cell types in 10 islets per case

Following image acquisition and analysis, sections were counterstained with haematoxylin to reveal islet histology and the adjacent exocrine region.

### Statistical analysis

A Student *t*-test in the excel of Microsoft Office 2016 was used for statistical analysis. Tests for significance were derived between two groups at a time only. *P* ≤ 0.05 was considered statistically significant.

## Results

### Immunohistochemical specificity of rabbit anti-GPX-1

In the immunohistochemical procedure for GPX-1, substitution of anti-rabbit GPX-1 with antibody diluent or normal rabbit IgG did not show staining of islets cells and the surrounding region (ESM Fig. 1 a-f). Incubation of a pancreatic section with rabbit anti-glucagon (also of rabbit origin as for GPX1 antibody employed in this study) in combination with guinea pig anti-insulin and mouse anti-CD45 (antibody of mouse origin as for glucagon) showed the expected staining of alpha cells, beta cells and CD45-positive cells, respectively (ESM Fig. 1 g-i). The addition of anti-GPX1 to separate pancreatic sections showed staining of several islet cells following incubation with anti-rabbit IgG horseradish peroxidase polymer and reaction with 3,3′diaminobenzidine (DAB) substrate (ESM Fig. 1 j-l) or donkey anti-rabbit IgG-Alexa 568 as secondary antibody (ESM Fig. 1 m–o).

Previous reports demonstrated that anti-GPX-1 (Abcam, ab22604; employed in this study) stained distinct cells in formalin-fixed tissue sections from lung carcinoma (Basnet et al. 2019). The corresponding transcript was also demonstrated in multiple tissues, including breast cancer (Chu et al. 1992; Woolston et al. 2011). In this study, we confirmed that anti-GPX1 stained cells in sections of formalin-fixed human lung, breast and, liver (ESM Fig. 2a–c). 

### Percentage of insulin-positive islets

Please refer to the demographic data for the characteristics of each donor in ESM Table 1.

**Fig. 3:**
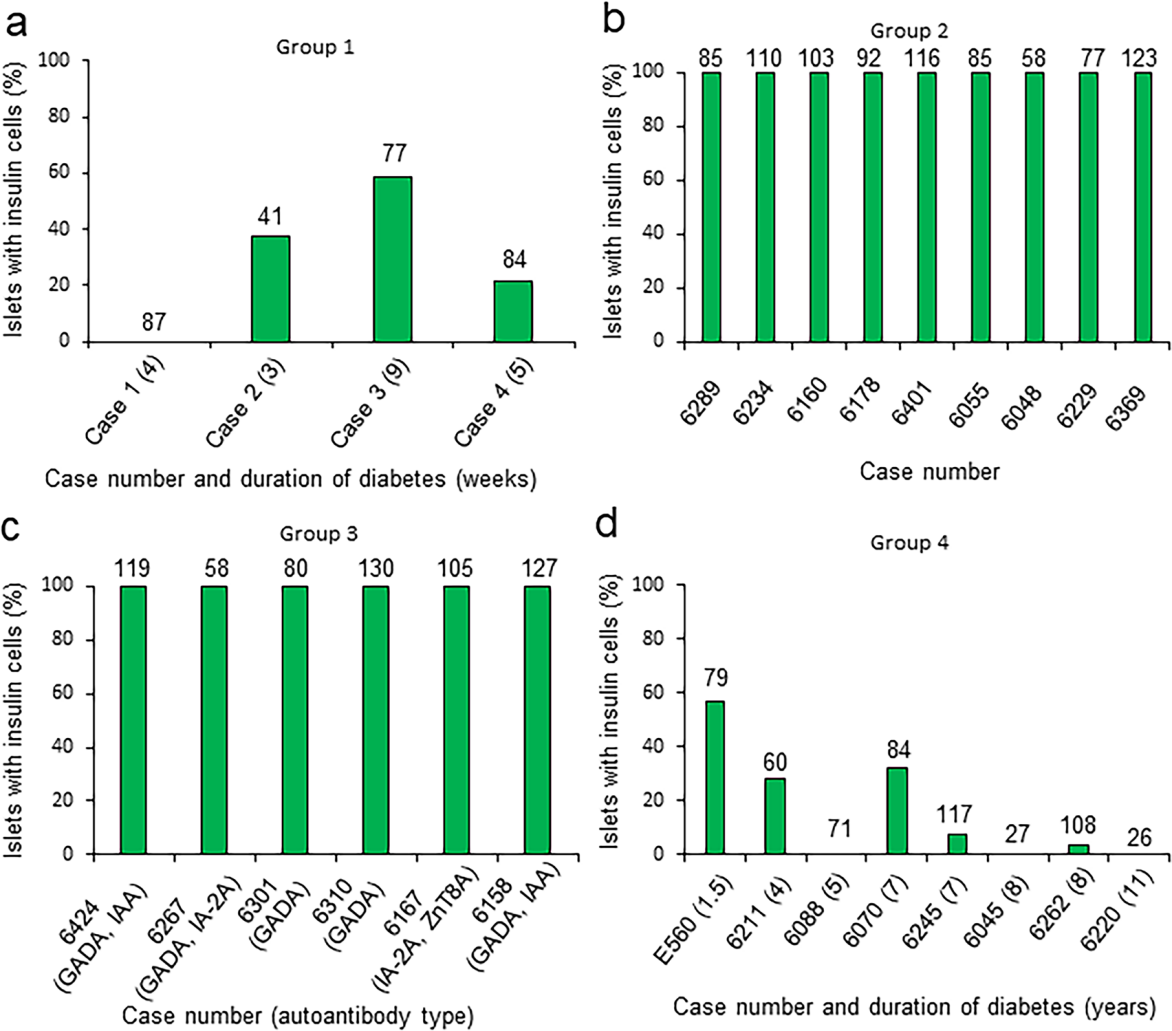
Percentage of insulin-positive islets (green bars) in pancreatic sections from donors belonging to groups 1 (**a**), 2 (**b**), 3 (**c**) and 4 (**d**). The total number of islets examined in each donor from groups 1 to 4 is indicated above each bar and in the second column of ESM Tables 3 (group 1), 4 (group 2), 5 (group 3) and 6 (group 4). In groups 1 and 4, the number within brackets adjacent to case idenities denotes duration of diabetes in weeks for group 1 and in years for group 4. GADA, anti-glutamic acid decarboxylase; IA-2A, anti-insulinoma associated antigen; IAA, insulin autoantibodies; ZnT8A, anti-zinc transporter 8

The percentage of insulin-positive islets per donor from the four study groups is shown in Fig. 3a–d (values per case are itemized in the second column of ESM Tables 3–6). In Fig. 3, the number of islets analyzed per case is indicated above each bar. In the non-diabetic autoantibody-negative and positive groups, all islets were insulin-positive (Fig. 3b, c). In Groups 1 and 4 (diabetic groups), 26.99% and 17.83% of islets were insulin-positive, respectively (Fig. 3a, d).

### Distribution and localization of GPX1 in islet cells

We observed inter-islet variability in the distribution and staining intensities of GPX1-positive islet cells in each donor and among different donors. Additional immunoreactivity was observed in selective pancreatic ductal cells, some of which were glucagon-positive (ESM Fig. 3).

In pancreatic islets, two major patterns of GPX1 expression were observed and defined as pattern 1 or 2 (Fig. 1a–f).

**Fig. 4:**
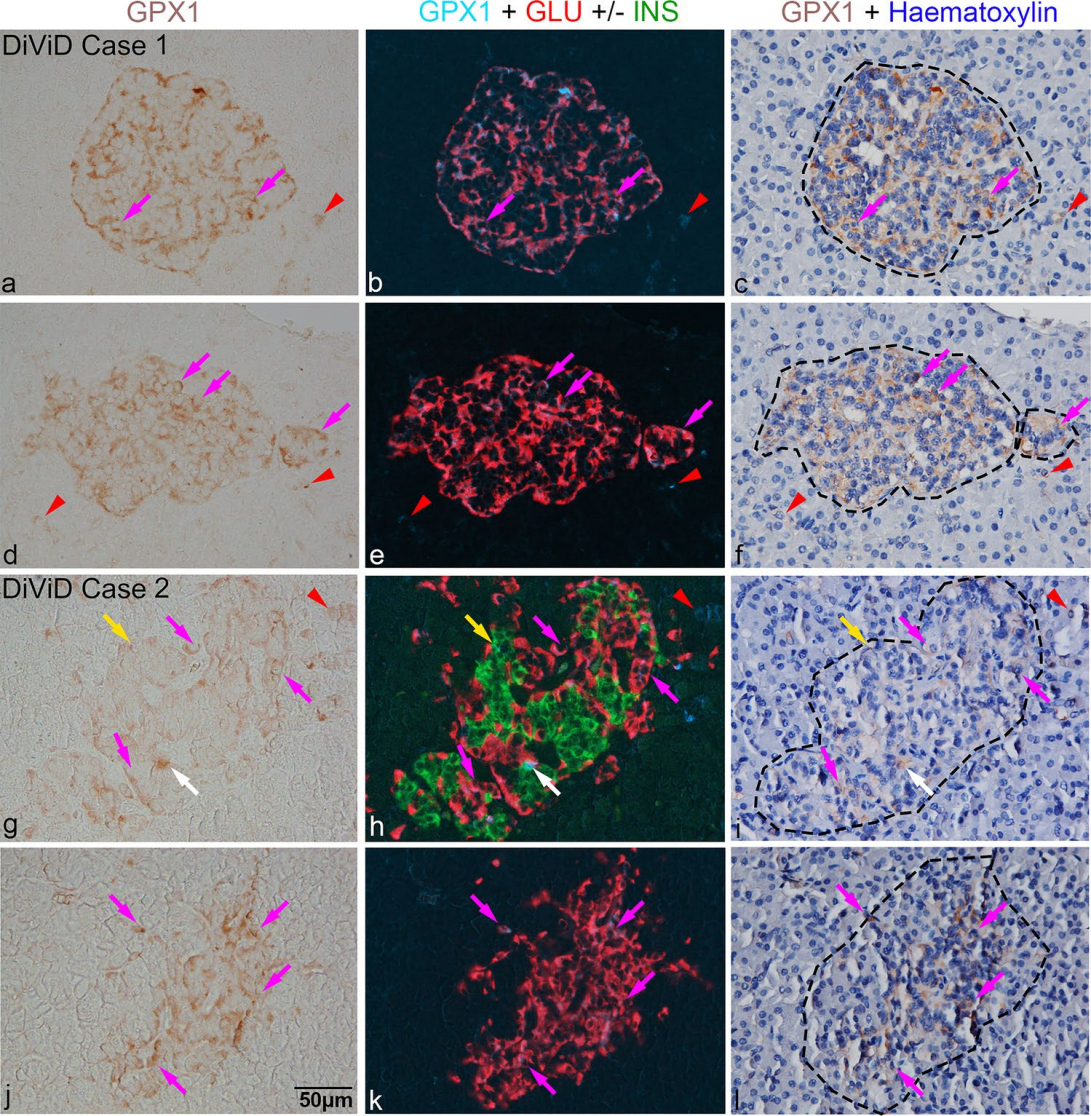
Immunohistochemical analysis of pancreas sections from DiViD donors 1 (**a**–**f**, diabetes duration 4 weeks) and 2 (**g**–**l**, diabetes duration 3 weeks). Left panel shows GPX1-positive cells (brown) in islets or occasionally outside the islet. The middle panel shows corresponding GPX1-positive cells in blue, merged with beta (green) and alpha (red) cells, following co-staining. The third panel shows corresponding fields after counterstaining with haematoxylin, where islet boundaries are indicated by black dashes. Magenta arrows indicate GPX1 in alpha cells and yellow arrows in beta cells while white arrows in cells negative for insulin and glucagon; red arrowheads indicate GPX1 staining in some insulin- and glucagon-negative cells in the exocrine region. Scale bar in (**j**), 50 μm, applies to all micrographs. DiViD, Diabetes Virus Detection Study; GLU, glucagon; GPX1, glutathione peroxidase-1; INS, insulin

**Fig. 5:**
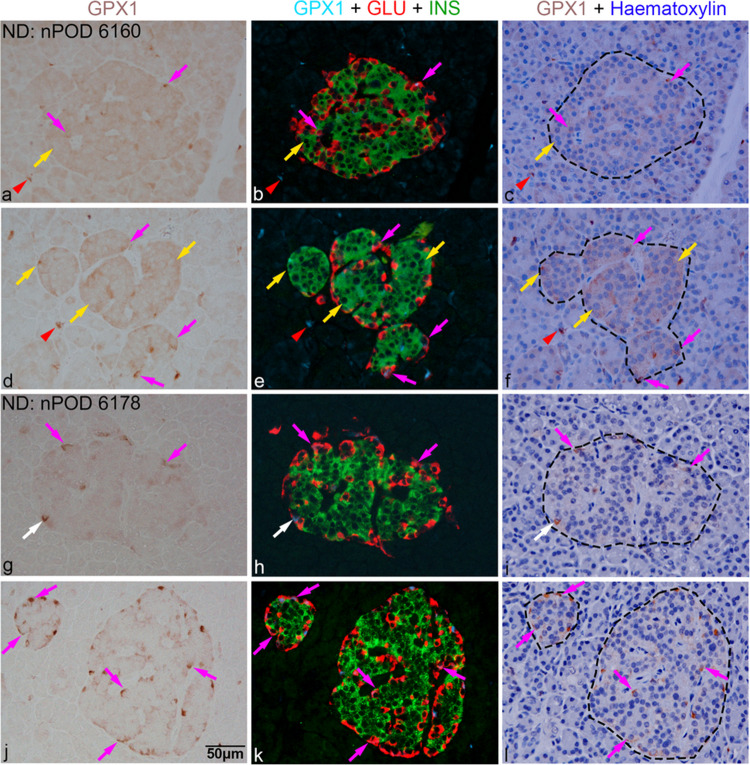
Immunohistochemical analysis of pancreas sections from non-diabetic autoantibody-negative nPOD donors 6160 (**a**–**f**) and 6178 (**g**–**l**). Left panel shows GPX1-positive cells (brown) in islets or occasionally outside the islet. The middle panel shows corresponding GPX1-positive cells in blue, merged with beta (green) and alpha (red) cells, following co-staining. The third panel shows corresponding fields after counterstaining with haematoxylin, where islet boundaries are indicated by black dashes. Magenta arrows indicate GPX1 in alpha cells and yellow arrows in beta cells while white arrows in cells negative for insulin and glucagon; red arrowheads indicate GPX1 staining in some insulin- and glucagon-negative cells in the exocrine region. Scale bar in (**j**), 50 µm, applies to all micrographs. GLU, glucagon; GPX1, glutathione peroxidase-1; INS, insulin; nPOD, Network for Pancreatic Organ Donors with Diabetes

**Fig. 6:**
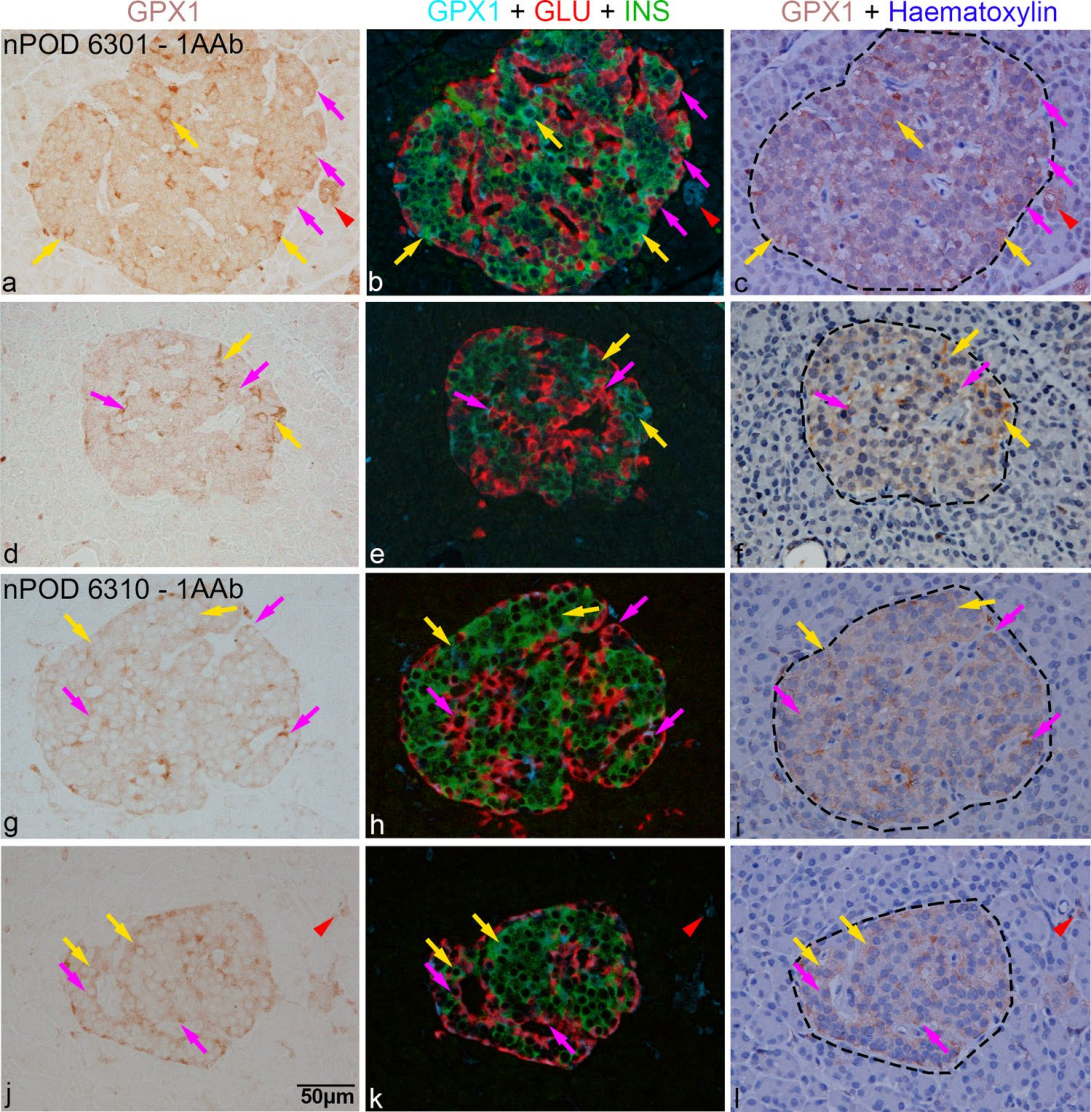
Immunohistochemical analysis of pancreas sections from non-diabetic autoantibody-positive nPOD donors 6301 (**a**–**f**) and 6310 (**g**–**l**). Left panel shows GPX1-positive cells (brown) in islets or occasionally outside the islet. The middle panel shows corresponding GPX1-positive cells in blue, merged with beta (green) and alpha (red) cells, following co-staining. The third panel shows corresponding fields after counterstaining with haematoxylin, where islet boundaries are indicated by black dashes. Magenta arrows indicate GPX1 in alpha cells and yellow arrows in beta cells; red arrowheads indicate GPX1 staining in some insulin- and glucagon-negative cells in the exocrine region. Scale bar in (**j**), 50 µm, applies to all micrographs. AAb, autoantibodies; GLU, glucagon; GPX1, glutathione peroxidase-1; INS, insulin; nPOD, Network for Pancreatic Organ Donors with Diabetes

**Fig. 7:**
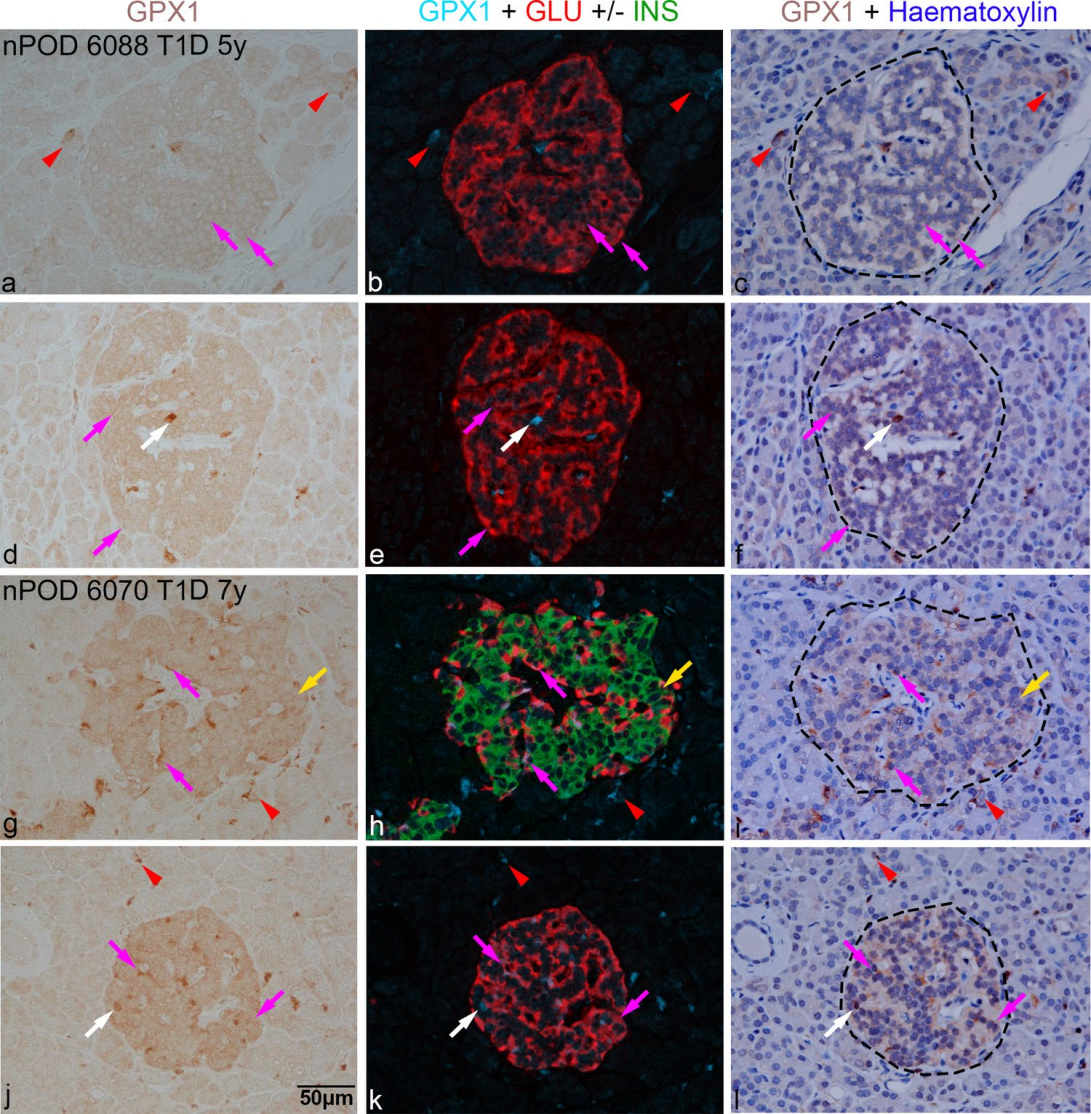
Immunohistochemical analysis of pancreas sections from long-term diabetic donors nPOD 6088 (**a**–**f**; diabetes duration, 5 years) and nPOD 6070 (**g**–**l**; diabetes duration, 7 years). Left panel shows GPX1-positive cells (brown) in islets or occasionally outside the islet. The middle panel shows corresponding GPX1-positive cells in blue, merged with beta (green) and alpha (red) cells, following co-staining. The third panel shows corresponding fields after counterstaining with haematoxylin, where islet boundaries are indicated by black dashes. Duration of diabetes in years is specified in (**a**, **g**). Magenta arrows indicate GPX1 in alpha cells and yellow arrows in beta cells while white arrows in cells negative for insulin and glucagon; red arrowheads indicate GPX1 staining in some insulin- and glucagon-negative cells in the exocrine region. Duration of diabetes in years is indicated in (**a**) and (**g**). Scale bar in (**j**), 50 µm, applies to all micrographs. GLU, glucagon; GPX1, glutathione peroxidase-1; INS, insulin; nPOD, Network for Pancreatic Organ Donors with Diabetes, T1D, type 1 diabetes

Examples of the overall distribution of GPX1-positive cells in selected islets from several donors are depicted as assembled micrographs, where the first column shows the presence of GPX1 cells alone within islets and the surrounding regions, the second column displays merged images of cells positive for GPX1 (brown GPX1 cells converted to a blue pseudo colour), insulin (green) and glucagon (red), while the third column shows corresponding fields after counterstaining with haematoxylin (Figs. 4, 5, 6, and 7; ESM Fig. 4–7). The expression and distribution of GPX1 cells in pancreatic islets were variable. GPX1 immunoreactivity in islet cells corresponded to staining patterns 1 (grades 1–3) and 2 (grades 4–6), irrespective of diabetes status.

### Analysis of overall GPX1 staining patterns in islets in the four study groups

**Fig. 8:**
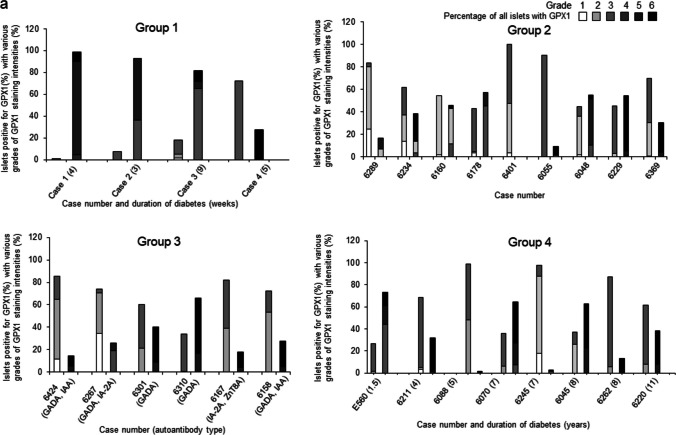

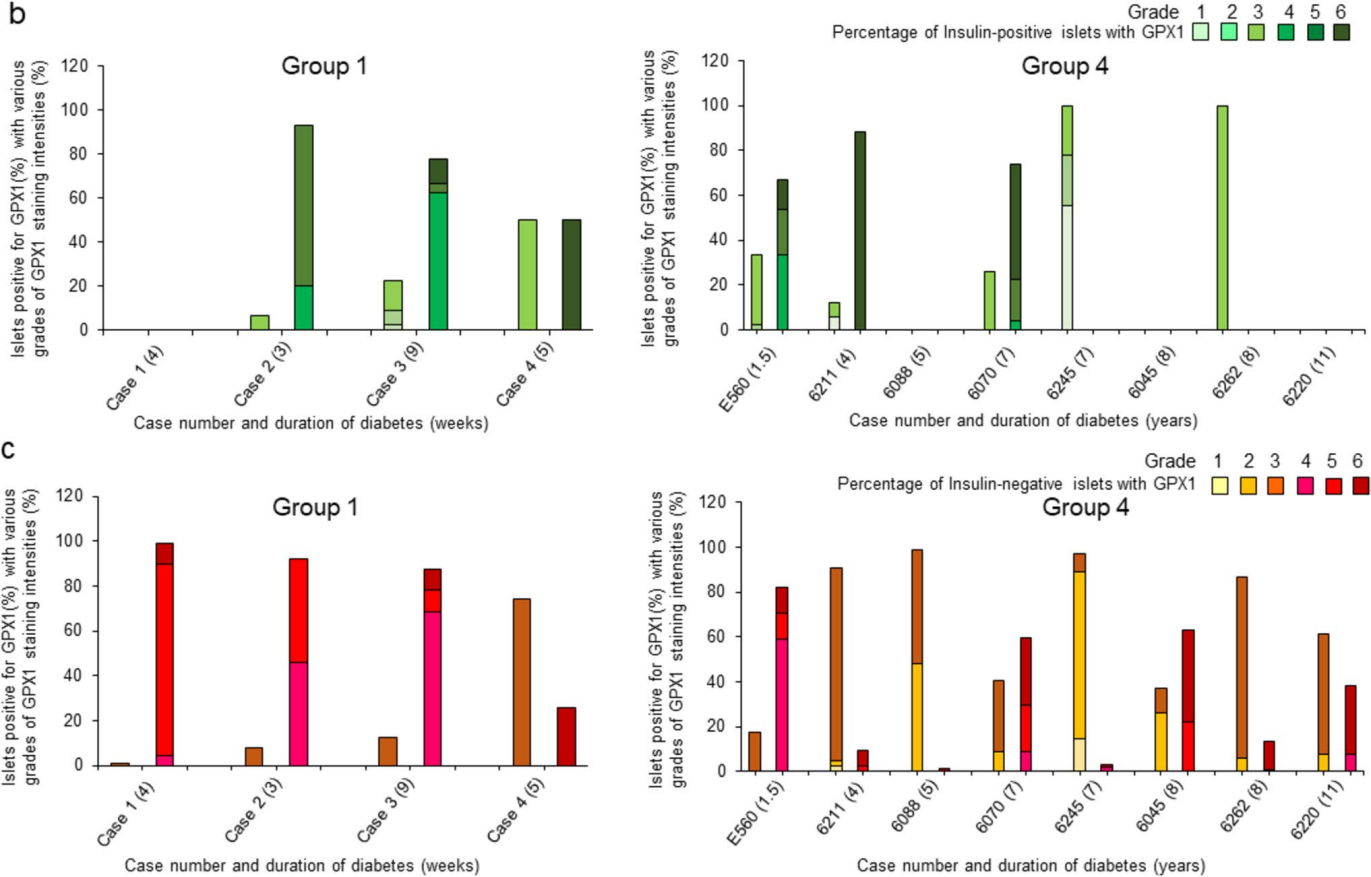
**a** Percentages of all islets per donor in the four study groups with graded staining intensities of GPX1 ranging from 1 to 3 (pattern 1) and 4 to 6 (pattern2) shown as adjacent bars for each case (refer to ESM Tables 3–6 for individual values). **b** Percentages of insulin-positive and insulin-negative islets with graded GPX1 staining intensities of 1–3 and 4–6 are shown as adjacent bars in diabetic cases (groups 1 and 4; individual values are shown in ESM Tables 7–10). In group 1 and 4, the number within brackets adjacent to case identities denotes duration of diabetes in weeks for group 1 and in years for group 4. GADA, anti-glutamic acid decarboxylase; IA-2A, anti-insulinoma associated antigen; IAA, insulin autoantibodies; ZnT8A, anti-zinc transporter 8

The overall cellular pattern of GPX1 expression in islets and its staining intensities in islet cells and insulin-negative and insulin-positive islets were quantified based on a visual grading system depicted in Fig. 1 (Pattern 1 (grades 1–3) and Pattern 2 (grades 4–6). The various grades in all islets are indicated as segmented values within each bar for each donor in Fig. 8a. Values for the percentages of islets with each grade per donor are listed in ESM Tables 3–6.

Gradings for GPX1 in insulin-positive and negative islets (groups 1 and 4) are represented as segmented bar graphs in Fig. 8b and tabulated in ESM Tables 7–10. In group 1 (newly diagnosed diabetic group), there was no significant difference between the percentage of insulin-positive islets with pattern 1 and pattern 2 (*p* = 0.09) and in insulin-negative islets between the same 2 patterns (*p* = 0.80). However, in group 4 (long-term diabetic group), there was a significant difference between the two patterns in insulin-positive islets (*p* = 0.04) and not in insulin-negative islets (*p* = 0.06).

### Analysis of GPX1 staining intensities in individual insulin and glucagon cells in 10 randomly selected islets per case from all study groups (refer to Fig. 9 and ESM Tables 11–14)

**Fig. 9:**
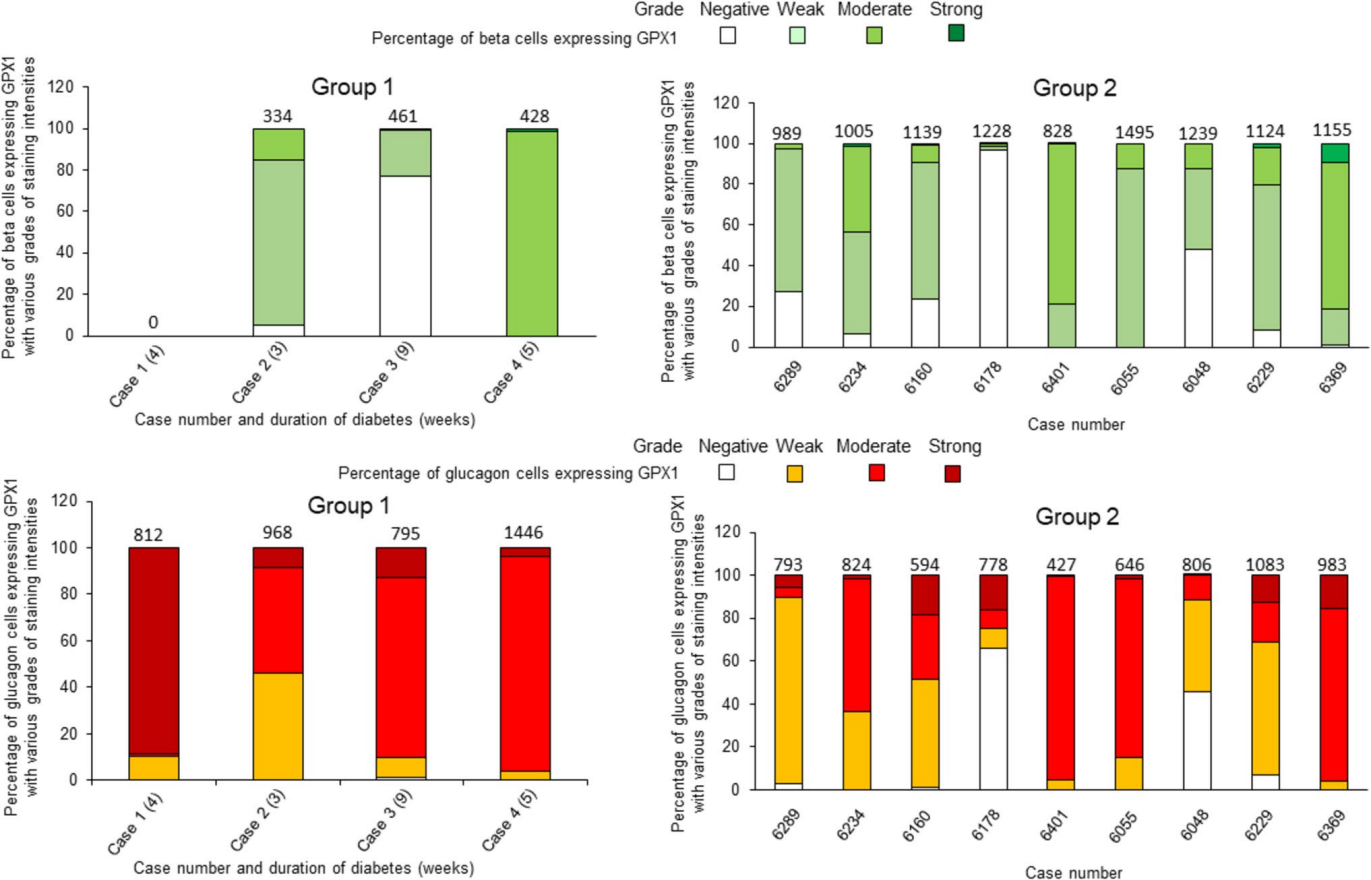

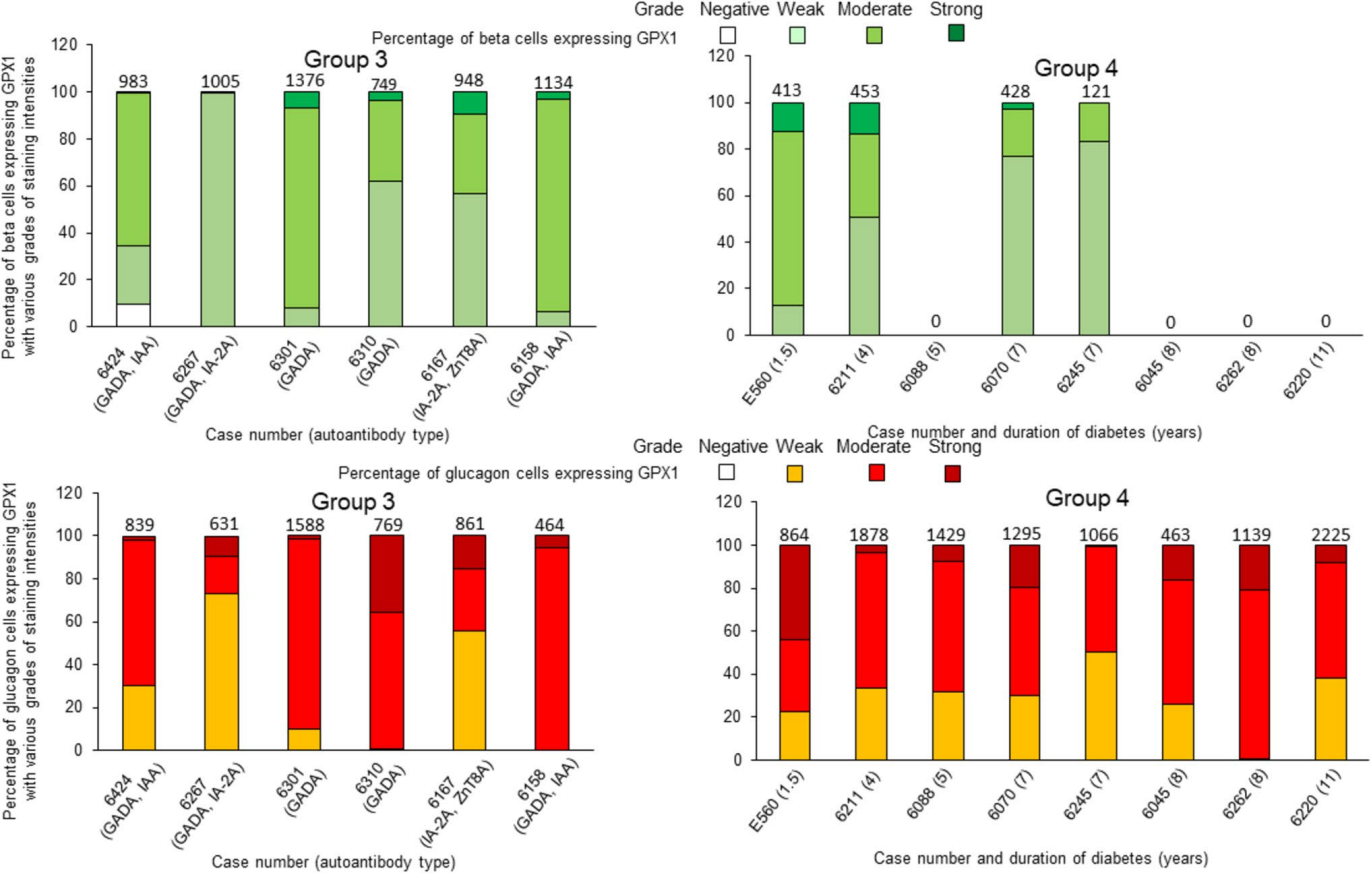
Percentages of beta cells and glucagon cells in 10 randomly selected islets per case showing GPX1 staining intensities graded on a scale of negative, weak, moderate and strong. Percentages are shown as segments within each bar (refer to ESM Tables 10–14 for individual values). Total number of insulin and glucagon cells analyzed for each case are shown above each bar. In group 1, the number within brackets adjacent to cases denotes duration of diabetes in weeks and in years for group 4. GADA, anti-glutamic acid decarboxylase; IA-2A, anti-insulinoma associated antigen; IAA, insulin autoantibodies; ZnT8A, anti-zinc transporter 8

#### Group 1 (newly diagnosed cases)

Islets from case 1 were insulin-negative, with strong GPX1 staining in approximately 90% of glucagon cells, while in case 2, 80% of beta cells had weak staining and almost 55% of glucagon cells had moderate to strong staining. In case 3, 77% of insulin cells had negative GPX1 staining, while almost 90% of glucagon cells had moderate to strong staining for GPX1. In case 4, almost all insulin and glucagon cells showed moderate GPX1 staining intensities.

#### Group 2 (non-diabetic autoantibody-negative cases)

In cases 6289, 6160, 6055 and 6229, many insulin cells showed weak GPX1 staining while in case 6178, 97% of such cells were negative with only 24% of glucagon cells with moderate to strong staining. In case 6401, > 78% of insulin cells had moderate GPX1 intensities. More than 80% of glucagon cells in cases 6401 and 6369 had moderate GPX1 intensity, while in the remaining cases, the intensities in the same cell types were more variable.

#### Group 3 (non-diabetic autoantibody-positive cases)

In cases 6424, 6301 and 6158, more than 65% of insulin cells showed moderate GPX1 staining intensities while in cases 6267, 6310 and 6167, 99%, 62% and 56%, respectively, showed weak intensities. In cases 6424, 6301, 6310 and 6158, moderate intensities were present in 68%, 88%, 64% and 94% of glucagon cells, respectively. In cases 6267 and 6167, weak intensities were present in 73% and 56% of glucagon cells, respectively. In all six cases, strong intensities were observed in a minority of glucagon cells (< 15% of glucagon cells), with staining intensities independent of single or double autoantibody status.

In groups 1, 2 and 3, differences between the percentages of insulin cells versus glucagon cells with negative, weak and moderate GPX1 staining intensities did not reach statistical difference.

However, there was a significant difference in Group 2 between insulin and glucagon cells with strong GPX1 staining (*p* = 0.03).

#### Group 4 (long-term diabetic cases)

Four of 8 cases from this group showed an absence of insulin cells in 10 randomly selected islets per case. In cases 6211, 6070 and 6245, more than 50% of insulin cells showed weak staining intensities for GPX1, while in case E560, approximately 75% of insulin cells displayed moderate staining intensities. Weak intensities were displayed in most of the remaining insulin cells (6211, 50.55%; 6070, 76.6%; 6245, 83.5%). In 7/8 cases from the same group, more than 50% of glucagon cells per case showed moderate staining intensities. In contrast, in the remaining case (E560: 1.5 years of T1D), 75% of glucagon cells showed moderate to strong intensities. In this group, there was a significant difference in the percentages of insulin cells versus glucagon cells with moderate to strong GPX1 staining intensities (*p* = 0.003).

### Cumulative percentages of graded GPX1 staining intensities in beta and glucagon cells per study group (refer to ESM Table 15 and Fig. 10)

**Fig. 10:**
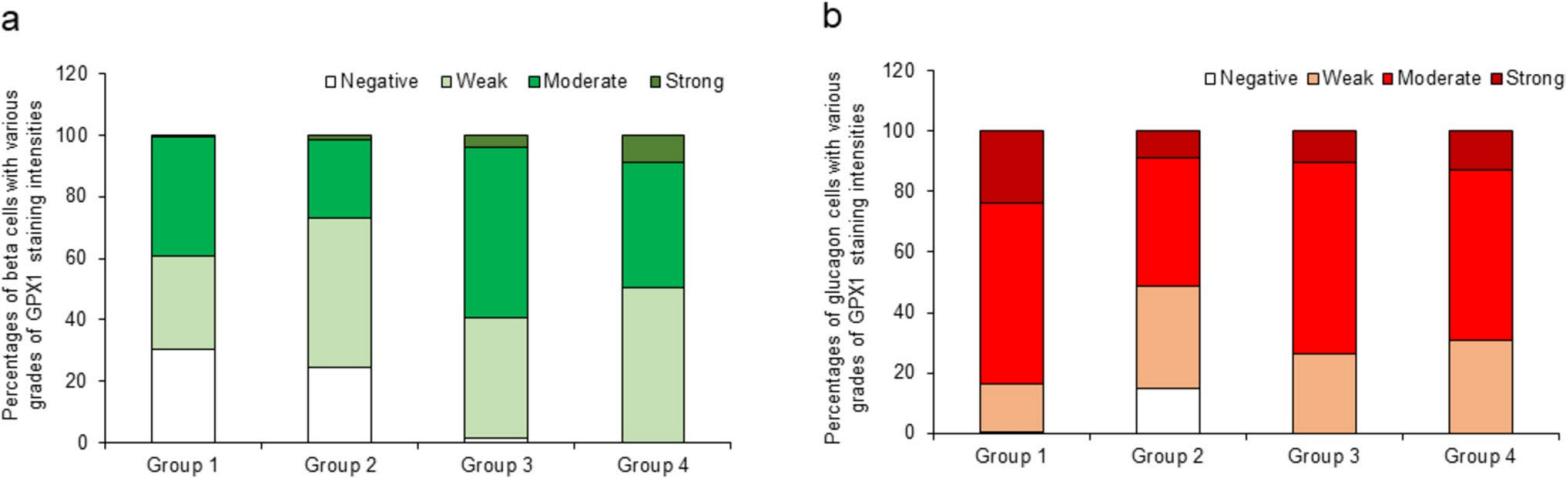
Cumulative percentages of beta cells (left bar graphs) and glucagon cells (right bar graphs) in the four study groups showing GPX1 staining intensities graded on a scale of negative, weak, moderate and strong. Percentages are shown as segments within each bar. Note: Cumulative percentages were calculated as a sum of all beta or glucagon cells in each study group with graded intensities. Ten randomly selected islets per case were analyzed. Refer to ESM Table 15 for cumulative percentages for each group

GPX1 staining in group 1 was absent in a higher percentage of insulin cells (30.34%) than glucagon cells (0.2%) (ESM Table 15). A similar difference was seen in groups 2 and 3, while in group 4 (long-term diabetic group), almost all insulin and glucagon cells showed weak to strong GPX1 staining intensities. In all four groups, moderate to strong intensities were observed in a higher percentage of glucagon cells than beta cells (ESM Table 15). In Group 4, there was a significant difference between the percentage of beta and glucagon cells with moderate GPX1 staining intensity (*p* = 0.005).

## Discussion

Earlier studies in experimental mice have shown an intrinsic absence or lower expression levels of the three important ROS-clearing enzymes, namely GPX1, catalase and SOD, in pancreatic islets (Grankvist et al. 1981; Harmon et al. 2009; Lenzen 2008; Tiedge et al. 1997; Tiedge et al. 1999). If such findings hold true for human islets, one may deduce that human beta cells may also fail to clear excess ROS efficiently. However, low expression levels of the three enzymes, specifically in beta cells of mice, have not been studied in detail. If such deficits exist in human beta cells, the resulting redox imbalance may activate critical immune signals in these cells before and during human T1D (Gerber and Rutter 2017; Leenders et al. 2021; Mallone et al. 2022; Newsholme et al. 2019; Robertson and Harmon 2006, 2007). Findings in rodents have led to the evaluation of several therapeutic approaches targeted at boosting levels of SOD and/or GPX1 or the use of low molecular weight mimetics to suppress excess ROS in beta cell lines and experimental animals afflicted with various pathologies (Bertera et al. 2003; Forman and Zhang 2021; Harmon et al. 2009; Mallone et al. 2022; Tiedge et al. 1999). Although such efforts have yielded little success, more recent ploys incorporating specific and targeted payload delivery systems and techniques for upregulating transcription factors that activate specific genes encoding anti-oxidant enzymes are in progress (Poprac et al. 2017).

The availability of rare pancreatic sections from clinically well-defined diabetic and non-diabetic donors has provided us with an impetus to explore the expression of GPX1 in islet cells of human subjects with and without T1D.

The specificity of anti-GPX1 was confirmed in this study by demonstrating positive staining in human liver, lung and breast carcinoma, omission of anti-GPX1 in the immunohistochemical procedure and the successful use of GPX1 antibody from the same supplier by others (Basnet et al. 2019, Chu et al. 1992; Miki et al. 2018). We also validated the expression of GPX1 in human pancreatic sections by employing an immunofluorescence technique. Staining of the enzyme in some ductal cells, a proportion of which co-localized with glucagon is novel. Glutathione peroxidase-1 immunoreactivity in insulin- and glucagon-negative islet cells, shown here, likely represents somatostatin and/or pancreatic polypeptide hormone and ghrelin cells or non-endocrine cells located within the intra-islet vascular region, such as endothelial cells or immune cells. Our results demonstrate that the distribution of GPX1 in the human pancreatic islet cells is more widespread than previously reported by others (Miki et al. 2018).

Our observation of two major cellular patterns of GPX1 immunoreactivity in islet cells (Pattern 1 and Pattern 2) has not been observed previously, and their physiological significance remains unclear. Different percentages of islets with the two staining patterns, with varying intensities, were observed in all donors, irrespective of diabetes status or the presence or absence of beta cells.

We demonstrate that various percentages of beta cells do express GPX1 in all study groups, with moderate to strong staining intensities in a higher percentage of glucagon cells than beta cells, except in nPOD case 6178, where a majority of insulin and glucagon cells were negative for GPX1. These observations suggest that in most cases, many beta cells express weaker or negative staining intensities for GPX1 than glucagon cells. We speculate that many beta cells may be ill-equipped to clear excess H_2_O_2_, conferring their oxidant vulnerability. Het-erogeneity of intraislet staining intensities in insulin and gluca-gon cells with islet-to-islet variability within the same donor may suggest differing rates of intracellular storage of GPX1 and its turnover within the same donor pancreas. Previous findings which show intra-islet functional heterogeneity, such as asynchronous secretion of insulin in response to glucose and beta cell senescence, lend support to such a suggestion (Farack et al. 2019; Thompson et al. 2023; Van Schravendijk et al. 1992).

However, more recent studies in mice suggest that beta cells are equipped with other anti-oxidant systems to scav-enge excess ROS, and in particular H_2_O_2_. These include var-ious isoforms of peroxiredoxin, thioredoxin and thioredoxin reductase (Stancill et al. 2019; Stancill and Corbett 2021). Whether such findings hold true for human islets remains to be proven. Of relevance, earlier studies have shown that human islets are more resistant to oxidant-generating beta cell toxins such as alloxan, streptozotocin, nitroprusside or cytokine-induced injury than mouse islets (Welsh et al. 1995). In the same studies, enzyme activities of catalase and SOD were significantly lower in islets from mice than in humans. High levels of thioredoxin-interacting protein (TXNIP) in beta cells may inhibit the ability of thioredoxin in the peroxiredoxin-thioredoxin system to detoxify H_2_O_2_ (Ovalle et al. 2018). The latter findings have led to an ongo-ing clinical trial in adults with recent-onset T1D, involving the use of verapamil, a known antihypertensive calcium channel blocker, in suppressing TXNIP levels and improving beta cell function (Ovalle et al. 2018).

We are cognizant of some limitations of the present study. For example, we have examined a small number of donor pan-creas and only analyzed the pancreatic tail region. Immuno-histochemistry, as the sole experimental approach, may have some limitations, such as sensitivity, but unlike Western blot-ting or proteomic analysis, it provides cellular identification and spatial distribution of immunoreactive cells without tissue disruption. The application of greater sensitive approaches, such as imaging mass spectrometry to decipher the expression of GPX1 in specific cell types more precisely, warrant future investigations (Prentice et al. 2019). Thus, our present immunohistochemical approach is compatible with the aims of the present study. We recognize that this study involved analysis of a smaller sample size due to the limited availability of rare human samples. Therefore, generalizations of our findings to all individuals with new-onset and long-term T1D require some caution. We recognize that while DiViD donors were in better diabetic control during and prior to biopsy, deceased donors from cadaveric and autopsy pancreas may have experi-enced variable degrees of unavoidable metabolic fluctuations, hyperglycaemia-driven oxidative and nitrosative stress and cold ischaemia before pancreas sampling. Prior medications during hospitalization, patient stress and variable but unavoid-able delays between death and tissue procurement may also be confoundable variables. Since our immunohistochemical approach does not discriminate between enzymatically active and inactive forms of GPX1, monomeric and tetrameric forms and gene variants, caution also needs to be exercised in ascrib-ing a physiological role of GPX1 solely based on immunohistochemical approaches (Mohammedi et al. 2016). We also recognize that although our studies are crosssectional and reflect a static snapshot of GPX1 expressing cells in human pancreatic sections, they are nevertheless informative and provide important clues on beta cell stress. Other limitations include the possibility of regional differences in the frequency, distribution and intensity of GPX1-positive cells within vari-ous islets from the same donor. Differences in the distribution of islets in the human pancreatic head, body and tail, with a selective loss of islets in the head region of donors with type 2 diabetes have been reported (Wang et al. 2013). Thus, the expression patterns of GPX1 in the three anatomical sites are yet to be determined. However, in this study, we report our results after carefully analyzing multiple islets with various crosssectional areas per donor. Future studies may provide more precise and mechanistic information on the distribution and vulnerability of human beta cells to excess levels of ROS, how they participate in early beta cell destruction and whether beta cells show protective mechanisms when exposed to raised levels of ROS.

The present study conducted in rare human pancreatic tissues, unveils scientific perspectives and offers new and previously unreported findings on the cellular patterns of GPX1 expression. Contrary to recent findings, we report that its expression is seen with varying intensities in not only in selective glucagon cells but also in beta cells in both non-diabetic and diabetic human donors. Unlike results from experimental mice, our current findings demonstrating GPX1 positivity in human beta cells are novel. Heterogeneous stain-ing of GPX1 in beta cells is consistent with recent obser-vations that demonstrate asynchronous destruction of beta cells during T1D (Thompson et al. 2023). Targeted delivery of GPX1 or its mimetics to beta cells at the early stages of T1D or pharmacological attempts to boost GPX-1 via the transcription factor, nuclear factor erythroid 2-related factor 2 (Nrf2) and other beta cell protective candidates may mitigate further destruction of beta cells (Forman and Zhang 2021).

## Supplementary Information

Below is the link to the electronic supplementary material.

Supplementary file1 (DOCX 38.0 KB )

Supplementary file2 (DOCX 20.4 KB)

Supplementary file3 (DOCX 42 KB)

Supplementary file4 (DOCX 35 KB)

Supplementary file5 (PDF 1.90 MB)

## Data Availability

All data supporting the findings of this study are available within the paper and its Supplementary Information.

## Declarations

### Disclaimer

The content and views expressed in this publication are the responsibility of the authors and do not reflect an official view of nPOD, DiViD and EADB.
