# Supplementary material for: Distribution of glutathione peroxidase-1 immunoreactive cells in pancreatic islets from type 1 diabetic donors and non-diabetic donors with and without islet cell autoantibodies is variable and independent of disease
Source: Cell Tissue Res. 2025 Mar 10;400(3):255–71. doi: 10.1007/s00441-025-03955-5 (PMC12125085; doi:10.1007/s00441-025-03955-5)
Supplement: Supplementary file 1 — Supplementary file1 (DOCX 38.0 KB ) [file 441_2025_3955_MOESM1_ESM.docx]

**ESM Table 1** Demographic information and case characteristics

| Case ID  and RRID for nPOD cases | Disease status and AAb  positivity | Duration of T1D (weeks or years) | Age at death/biopsy (years), sex, ethnicity, BMI | Autoantibody type | C-peptide (ng/mL) | Cause of death (relevant history and pancreatic disorders – nPOD and EADB cases only) |
| --- | --- | --- | --- | --- | --- | --- |
| *Group 1: Newly-diagnosed cases (DiViD)* | | | | | | |
| Case 1 | T1D, 4AAb | 4 weeks | 25, F, white, 21.0 | GADA, IA-2A, IAA, ZnT8A | 1.11 | Live donor |
| Case 2 | T1D, 3AAb | 3 weeks | 24, M, white, 20.9 | GADA, IA-2A, ZnT8A | 1.05 | Live donor |
| Case 3 | T1D, 3AAb | 9 weeks | 34, F, white, 23.7 | GADA, IA-2A, ZnT8A | 2.4 | Live donor |
| Case 4 | T1D, 3AAb | 5 weeks | 31, M, white, 25.6 | GADA, IA-2A, IAA | NA | Live donor |
|  |  |  |  |  |  |  |
| *Group 2: Non-diabetic AAb-negative cases (nPOD & EADB)* | | | | | | |
| 6289  (SAMN15879343) | Non-diabetic | - | 19, M, Af-Am, 38.3 | - | 8.05 | Head trauma |
| 6234  (SAMN15879290) | Non-diabetic | - | 20, F, white, 25.6 | - | 6.89 | Head trauma |
| 6160  (SAMN15879216) | Non-diabetic | - | 22.1, M, white, 23.9 | - | 0.4 | Head trauma |
| 6178  (SAMN15879234) | Non-diabetic | - | 24.5, F, white, 27.5 | - | 4.55 | Anoxia |
| 6401  (SAMN15879454) | Non-diabetic | - | 25.1, F, Hispanic, 31.3 | - | 12.81 | Head trauma |
| 6055  (SAMN15879112) | Non-diabetic | - | 27, M, white, 22.7 | - | 0.59 | Anoxia |
| 6048  (SAMN15879105) | Non-diabetic | - | 30, M, white, 20.6 | - | 17.91 | Cerebrovascular/stroke |
| 6229  (SAMN15879285) | Non-diabetic | - | 31, F, white, 26.9 | - | 6.23 | Head trauma |
| 6369  (SAMN15879422) | Non-diabetic | - | 44, M, white, 18.8 | - | 6.42 | Cerebrovascular/stroke |
|  |  |  |  |  |  |  |
| *Group 3: Non-diabetic AAb-positive cases (nPOD)* | | | | | | |
| 6424  (SAMN15879477) | Non-diabetic, 2AAb | - | 17.65, M, white, 51.4 | GADA, IAA | 6.97 | Head trauma |
| 6267  (SAMN15879321) | Non-diabetic, 2AAb | - | 23, F, white, 23.5 | GADA, IA-2A | 16.59 | Anoxia |
| 6301  (SAMN15879355) | Non-diabetic, 1AAb | - | 26, M, Af-Am, 32.1 | GADA | 3.92 | Head trauma |
| 6310  (SAMN15879364) | Non-diabetic, 1AAb | - | 28, F, Hispanic, 22.4 | GADA | 10.54 | Anoxia; low grade insulitis |
| 6167  (SAMN15879223) | Non-diabetic, 2AAb | - | 37, M, white, 26.3 | IA-2A, ZnT8A | 5.43 | Head trauma |
| 6158  (SAMN15879214) | Non-diabetic, 2AAb | - | 40.3, M, white, 29.7 | GADA, IAA | 0.51 | Head trauma |
| *Group 4: Diabetic cases (nPOD and EADB)* | | | | | | |
| E560 | T1D | 1.5 years | 42, F, ethnicity NA, BMI NA | NA | NA | NA |
| 6211  (SAMN15879267) | T1D, 4AAb | 4 years | 24, F, Af-Am, BMI NA | GADA, IA-2A, IAA, ZnT8A | <0.05 | Anoxia; Very mild chronic inflammation within interstitial fibrosis and mononuclear infiltration (2 foci) |
| 6088  (SAMN15879145) | T1D, 4AAb | 5 years | 31.2, M, white, 27.0 | GADA, IA-2A, IAA, ZnT8A | <0.05 | Head trauma; mild, chronic pancreatitis, insulitis |
| 6070  (SAMN15879127) | T1D, 2AAb | 7 years | 22.6, F, white, 21.6 | IA-2A, IAA | <0.05 | Anoxia |
| 6245  (SAMN15879301) | T1D, 2AAb | 7 years | 22, M, white, 23.2 | GADA, IA-2A | <0.05 | Head trauma; very mild chronic inflammation, insulitis- sparse, multiple hospitalizations with DKA |
| 6045  (SAMN15879102) | T1D, 2AAb | 8 years | 26.4, M, white, 23.1 | IAA, ZnT8A | <0.05 | Head trauma |
| 6262  (SAMN15879316) | T1D, 3AAb | 8 years | 44, M, Af-Am, 21.5 | GADA, IA-2A, IAA | <0.05 | Anoxia; very mild chronic inflammation |
| 6220  (SAMN15879276) | T1D, 2AAb | 11 years | 35, F, white, 27.4 | GADA, IAA | <0.05 | Anoxia |

Abbreviations: AAb, autoantibody; Af-Am, African American; BMI, body mass index (kg/m^2^); DKA, diabetic ketoacidosis; EADB, Exeter Archival Diabetes Biobank; F, female; GADA, anti-glutamic acid decarboxylase; IA-2A, anti-insulinoma-associated antigen; IAA, insulin autoantibodies; M, male; NA, not available; T1D, type 1 diabetes; ZnT8A, anti-zinc transporter 8

Cases 1-4 were from the DiViD Study where sections were prepared from pancreatic biopsies from living donors with recent onset T1D. All nPOD sections, were from cadaveric pancreas. Note: Case 6424 has BMI 51.4, an outlier from the rest of the cases selected, but included due to his non-diabetic autoantibody positive status. Sections from Case E560 (group 4), were from autopsy samples supplied by EADB.

Each nPOD case has a unique Research Resource Identifier (RRID) beginning with “SAMN” which is specified in the first column within brackets.
