# Supplementary material for: Distribution of glutathione peroxidase-1 immunoreactive cells in pancreatic islets from type 1 diabetic donors and non-diabetic donors with and without islet cell autoantibodies is variable and independent of disease
Source: Cell Tissue Res. 2025 Mar 10;400(3):255–71. doi: 10.1007/s00441-025-03955-5 (PMC12125085; doi:10.1007/s00441-025-03955-5)
Supplement: Supplementary file 2 — Supplementary file2 (DOCX 20.4 KB) [file 441_2025_3955_MOESM2_ESM.docx]

**ESM Table 2.** List of primary antibodies, conjugated secondary antibodies linked to Alexa dyes and Dylight 488 employed in combined immunohistochemical protocol for GPX1, insulin and glucagon. Also included are suppliers, dilutions, diluent and incubation period during immunohistochemistry.

| **Antibodies/Reagents** | **Supplier** | **Catalogue number** | **Working Dilution** | **Diluent** | | **Incubation time; temperature** |
| --- | --- | --- | --- | --- | --- | --- |
| **Primary antibodies** |  |  |  |  | |  |
| Rabbit anti-GPX1 | Abcam | ab22604 | 1:120 | Cell Signaling Technology antibody diluent (catalogue number 8112S) | | 18 h, 4°C |
| Guinea pig anti-insulin | Dako, Glostrup, Denmark | AO564 | 1:600 | Cell Signaling Technology antibody diluent | | 1.5 h, 37°C |
| Mouse anti-glucagon | Sigma Aldrich, Darmstadt,  Germany | SAB4200685 | 1:600 | Cell Signaling Technology antibody diluent | | 1.5 h, 37°C |
| Rabbit anti-glucagon | Dako, Glostrup, Denmark | AO565 | 1:200 | Cell Signaling Technology antibody diluent | | 18 h, 4ºC |
| Mouse anti-CD45 | Dako, Glostrup, Denmark | AO565 | 1:100 | Cell Signaling Technology antibody diluent | | 18 h, 4ºC |
| **Secondary antibodies** |  |  |  |  |  |  |
| Anti-rabbit IgG HRP polymer | Cell Signalling Technology,  Danvers, MA 02123, USA | 8114 | 1:1 | 0.1% vol./vol. Tween 20 in PBS | | 30 min, RT |
| Donkey anti-guinea pig IgG Alexa 488 | Jackson Immunoresearch; Westgrove, PA, USA | 706-545-148 | 1:400 | 0.2% vol./vol. Tween 20 in PBS | | 1 h, 37°C |
| Donkey anti-mouse IgG Alexa 568 | Invitrogen; Eugene, OR, USA | A10037 | 1:600 | 0.2% vol./vol. Tween 20 in PBS | | 1 h, 37°C |
| Donkey anti-rabbit IgG (H+L) Alexa 568 | Invitrogen; Eugene, OR,  USA | A10042 | 1:400 | 0.2% vol./vol. Tween20 in PBS | | 1 h, 37°C |
| Horse anti-mouse IgG (H+L) Dylight 488 | Vector, Newark, CA,  USA | DE-2488-1.5 | 1:200 | 0.2% vol./vol. Twen20 in PBS | | 1 h, 37°C |

RT, room temperature; PBS, phosphate-buffered saline; vol./vol., volume/volume; h, hour/s; min, minute/s.

Guinea pig anti-insulin from Dako and mouse anti-glucagon from Sigma have been validated for immunohistochemistry in this laboratory. Rabbit anti-GPX1 from Abcam has been previously verified for use in immunohistochemistry on formalin-fixed human tissue sections and also in this laboratory with formalin-fixed human tissue sections from breast, liver and lung cancer. Negative controls for GPX1 immunostaining were performed with omission of primary antibody and substituting with either diluent or normal rabbit IgG. Following completion of GPX1staining by immunoperoxidase, guinea pig anti-insulin and mouse anti-glucagon were applied as a mixture, followed by co-incubation with a mixture of donkey anti-guinea pig IgG-Alexa 488 and donkey anti-mouse IgG-Alexa 568. In control studies, horse anti-mouse IgG-Dylight 488 was also employed to reveal glucagon immunoreactive cells.
