# Supplementary material for: Distribution of glutathione peroxidase-1 immunoreactive cells in pancreatic islets from type 1 diabetic donors and non-diabetic donors with and without islet cell autoantibodies is variable and independent of disease
Source: Cell Tissue Res. 2025 Mar 10;400(3):255–71. doi: 10.1007/s00441-025-03955-5 (PMC12125085; doi:10.1007/s00441-025-03955-5)
Supplement: Supplementary file 3 — Supplementary file3 (DOCX 42 KB) [file 441_2025_3955_MOESM3_ESM.docx]

**ESM Table 3.** Group 1 (newly-diagnosed cases): Summary of percentages of islets positive for beta cells and with various grades of GPX1 staining intensities

| Case number and duration of diabetes from diagnosis in weeks | Total number of islets examined and (number of insulin-positive islets; percent insulin-positive) | Percentage of islets with GPX1 staining intensity of 1 (and number of islets) | Percentage of islets with GPX1 staining intensity of 2 (and number of islets) | Percentage of islets with GPX1 staining intensity of 3 (and number of islets) | Percentage of islets with GPX1 staining intensity of 4 (and number of islets) | Percentage of islets with GPX1 staining intensity of 5 (and number of islets) | Percentage of islets with GPX1 staining intensity of 6 (and number of islets) |
| --- | --- | --- | --- | --- | --- | --- | --- |
|  |  |  |  |  |  |  |  |
| DiViD case 1, 4 weeks | 87 (0; 0.00%) | 0.00% (0) | 0.00% (0) | 1.15% (1) | 4.60% (4) | 85.06% (74) | 9.20% (8) |
| DiViD case 2, 3 weeks | 41 (15; 36.59%) | 0.00% (0) | 0.00% (0) | 7.32% (3) | 36.59% (15) | 56.10% (23) | 0.00% (0) |
| DiViD case 3, 9 weeks | 77 (45; 58.44%) | 1.30% (1) | 3.90% (3) | 12.99% (10) | 64.94% (50) | 6.49% (5) | 10.39% (8) |
| DiViD case 4, 5 weeks | 84 (18; 21.43%) | 0.00% (0) | 1.19% (1) | 64.29% (54) | 0.00% (0) | 0.00% (0) | 34.52% (29) |

**ESM Table 4.** Group 2 (non-diabetic autoantibody-negative cases): Summary of percentages of islets positive for beta cells and with various grades of GPX1 staining intensities

| Case number | Total number of islets examined and (number of insulin-positive islets; percent insulin-positive) | Percentage of islets with GPX1 staining intensity of 1 (and number of islets) | Percentage of islets with GPX1 staining intensity of 2 (and number of islets) | Percentage of islets with GPX1 staining intensity of 3 (and number of islets) | Percentage of islets with GPX1 staining intensity of 4 (and number of islets) | Percentage of islets with GPX1 staining intensity of 5 (and number of islets) | Percentage of islets with GPX1 staining intensity of 6 (and number of islets) |
| --- | --- | --- | --- | --- | --- | --- | --- |
|  |  |  |  |  |  |  |  |
| 6289 | 85 (85; 100%) | 24.71% (21) | 55.29% (47) | 3.53% (3) | 0.00% (0) | 7.06% (6) | 9.41% (8) |
| 6234 | 110 (110; 100%) | 13.64% (15) | 23.64% (26) | 24.54% (27) | 3.64% (4) | 10.00% (11) | 24.54% (27) |
| 6160 | 103 (103; 100%) | 1.94% (2) | 52.43% (54) | 0.00% (0) | 11.65% (12) | 31.07% (32) | 2.91% (3) |
| 6178 | 92 (92; 100%) | 3.30% (3) | 1.09% (1) | 38.46 (35) | 45.05% (41) | 12.09% (11) | 0.00% (0) |
| 6401 | 116 (116; 100%) | 3.45% (4) | 43.96% (51) | 52.59% (61) | 0.00% (0) | 0.00% (0) | 0.00% (0) |
| 6055 | 85 (85; 100%) | 0.00% (0) | 0.00% (0) | 90.59% (77) | 0.00% (0) | 1.18% (1) | 8.24% (7) |
| 6048 | 58 (58 (100%) | 1.72% (1) | 34.48% (20) | 8.62% (5) | 10.34% (6) | 41.38% (24) | 3.45% (2) |
| 6229 | 77 (77; 100%) | 0.00% (0) | 2.60% (2) | 42.86% (33) | 1.30% (1) | 3.90% (3) | 49.35% (38) |
| 6369 | 123 (123; 100%) | 0.81% (1) | 29.27% (36) | 39.84% (49) | 0.00% (0) | 4.07% (5) | 26.02% (35) |

**ESM Table 5.** Group 3 (non-diabetic autoantibody-positive cases): Summary of percentages of islets positive for beta cells and with various grades of GPX1 staining intensities

| Case number | Total number of islets examined and (number of insulin-positive islets; percent insulin-positive) | Percentage of islets with GPX1 staining intensity of 1 (and number of islets) | Percentage of islets with GPX1 staining intensity of 2 (and number of islets) | Percentage of islets with GPX1 staining intensity of 3 (and number of islets) | Percentage of islets with GPX1 staining intensity of 4 (and number of islets) | Percentage of islets with GPX1 staining intensity of 5 (and number of islets) | Percentage of islets with GPX1 staining intensity of 6 (and number of islets) |
| --- | --- | --- | --- | --- | --- | --- | --- |
|  |  |  |  |  |  |  |  |
| 6424 | 119 (119; 100%) | 11.76% (14) | 52.94% (63) | 21.01% (25) | 0.84% (1) | 4.20% (5) | 9.24% (11) |
| 6267 | 58 (58; 100%) | 34.48% (20) | 36.21% (21) | 3.45% (2) | 18.97% (11) | 6.90% (4) | 0.00% (0) |
| 6301 | 80 (80; 100%) | 0.00% (0) | 21.25% (17) | 38.75% (31) | 0.00% (0) | 8.75% (7) | 31.25% (25) |
| 6310 | 130 (130; 100%) | 0.00% (0) | 0.77% (1) | 33.08% (43) | 0.00% (0) | 16.92% (22) | 49.23% (64) |
| 6167 | 105 (105; 100%) | 0.00% (0) | 39.05% (41) | 42.86% (45) | 0.00% (0) | 3.81% (4) | 14.29% (15) |
| 6158 | 127 (127; 100%) | 0.00% (0) | 53.54% (68) | 18.90% (24) | 0.79% (1) | 3.94% (5) | 22.83% (29) |

**ESM Table 6.** Group 4 (long-term diabetic cases): Summary of percentages of islets positive for beta cells and with various grades of GPX1 staining intensities

| Case number and duration of diabetes from diagnosis in years | Total number of islets examined and (number of insulin-positive islets; percent insulin-positive) | Percentage of islets with GPX1 staining intensity of 1 (and number of islets) | Percentage of islets with GPX1 staining intensity of 2 (and number of islets) | Percentage of islets with GPX1 staining intensity of 3 (and number of islets) | Percentage of islets with GPX1 staining intensity of 4 (and number of islets) | Percentage of islets with GPX1 staining intensity of 5 (and number of islets) | Percentage of islets with GPX1 staining intensity of 6 (and number of islets) |
| --- | --- | --- | --- | --- | --- | --- | --- |
|  |  |  |  |  |  |  |  |
| E560, 1.5 years | 79 (45; 56.96%) | 0.00% (0) | 1.27% (1) | 25.32% (20) | 44.30% (35) | 16.46% (13) | 12.66% (10) |
| 6211, 4 years | 60 (17; 21.25%) | 3.33% (2) | 1.67% (1) | 63.33% (38) | 0.00% (0) | 1.67% (1) | 30.00% (18) |
| 6088, 5 years | 71 (0; 0.00%) | 0.00% (0) | 47.89% (36) | 50.70% (34) | 0.00% (0) | 0.00% (0) | 1.41% (1) |
| 6070, 7 years | 84 (27; 32.14%) | 0.00% (0) | 5.95% (5) | 29.76% (25) | 7.14% (6) | 20.24% (17) | 36.90% (31) |
| 6245, 7 years | 117 (9; 7.69%) | 17.95% (21) | 70.09% (82) | 9.40% (11) | 1.71% (2) | 0.00% (0) | 0.85% (1) |
| 6045, 8 years | 27 (0; 0.00%) | 0.00% (0) | 25.93% (7) | 11.11% (3) | 0.00% (0) | 22.22% (6) | 40.74% (11) |
| 6262, 8 years | 108 (4; 3.70%) | 0.00% (0) | 5.56% (6) | 81.48% (88) | 0.00% (0) | 0.93% (1) | 12.04% (13) |
| 6220, 11 years | 26 (0; 0.00%) | 0.00% (0) | 7.69% (2) | 53.85% (14) | 0.00% (0) | 7.69% (2) | 30.77% (8) |

**ESM Table 7.** Group 1 (newly-diagnosed cases): Summary of number of insulin-positive islets and percentages of islets with various grades of GPX1 staining intensities

| Case number and duration of diabetes from diagnosis in weeks | Total number of insulin-positive islets | Percentage of insulin-positive islets with GPX1 staining intensity of 1 (and number of insulin-positive islets) | Percentage of insulin-positive islets with GPX1 staining intensity of 2 (and number of insulin-positive islets) | Percentage of insulin-positive islets with GPX1 staining intensity of 3 (and number of insulin-positive islets) | Percentage of insulin-positive islets with GPX1 staining intensity of 4 (and number of insulin-positive islets) | Percentage of insulin-positive islets with GPX1 staining intensity of 5 (and number of insulin-positive islets) | Percentage of insulin-positive islets with GPX1 staining intensity of 6 (and number of insulin-positive islets) |
| --- | --- | --- | --- | --- | --- | --- | --- |
|  |  |  |  |  |  |  |  |
| DiViD case 1, 4 weeks | 0 | 0.00% (0) | 0.00% (0) | 0.00% (0) | 0.00% (0) | 0.00% (0) | 0.00% (0) |
| DiViD case 2, 3 weeks | 15 | 0.00% (0) | 0.00% (0) | 6.67% (1) | 20.00% (3) | 73.33% (11) | 0.00% (0) |
| DiViD case 3, 9 weeks | 45 | 2.22% (1) | 6.67% (3) | 13.33% (6) | 62.22% (28) | 4.44% (2) | 11.11% (5) |
| DiViD case 4, 5 weeks | 18 | 0.00% (0) | 0.00% (0) | 61.11% (11) | 0.00% (0) | 0.00% (0) | 38.89% (7) |

**ESM Table 8.** Group 4 (long-term diabetic cases): Summary of number of insulin-positive islets and percentages of islets with various grades of GPX1 staining intensities

| Case number and duration of diabetes from diagnosis in years | Total number of insulin-positive islets | Percentage of insulin-positive islets with GPX1 staining intensity of 1 (and number of insulin-positive islets) | Percentage of insulin-positive islets with GPX1 staining intensity of 2 (and number of insulin-positive islets) | Percentage of insulin-positive islets with GPX1 staining intensity of 3 (and number of insulin-positive islets) | Percentage of insulin-positive islets with GPX1 staining intensity of 4 (and number of insulin-positive islets) | Percentage of insulin-positive islets with GPX1 staining intensity of 5 (and number of insulin-positive islets) | Percentage of insulin-positive islets with GPX1 staining intensity of 6 (and number of insulin-positive islets) |
| --- | --- | --- | --- | --- | --- | --- | --- |
|  |  |  |  |  |  |  |  |
| E560, 1.5 years | 45 | 0.00% (0) | 2.22% (1) | 31.11% (14) | 33.33% (15) | 20.00% (9) | 13.33% (6) |
| 6211, 4 years | 17 | 5.88% (1) | 0.00% (0) | 5.88% (1) | 0.00% (0) | 0.00% (0) | 88.24% (15) |
| 6088, 5 years | 0 | 0.00% (0) | 0.00% (0) | 0.00% (0) | 0.00% (0) | 0.00% (0) | 0.00% (0) |
| 6070, 7 years | 27 | 0.00% (0) | 0.00% (0) | 25.93% (7) | 3.70% (1) | 18.52% (5) | 51.85% (14) |
| 6245, 7 years | 9 | 55.56% (5) | 22.22% (2) | 22.22% (2) | 0.00% (0) | 0.00% (0) | 0.00% (0) |
| 6045, 8 years | 0 | 0.00% (0) | 0.00% (0) | 0.00% (0) | 0.00% (0) | 0.00% (0) | 0.00% (0) |
| 6262, 8 years | 4 | 0.00% (0) | 0.00% (0) | 100.00% (4) | 0.00% (0) | 0.00% (0) | 0.00% (0) |
| 6220, 11 years | 0 | 0.00% (0) | 0.00% (0) | 0.00% (0) | 0.00% (0) | 0.00% (0) | 0.00% (0) |

**ESM Table 9.** Group 1 (newly-diagnosed cases): Summary of number of insulin-negative islets and percentages of islets with various grades of GPX1 staining intensities

| Case number and duration of diabetes from diagnosis in weeks | Total number of insulin-negative islets | Percentage of insulin-negative islets with GPX1 staining intensity of 1 (and number of insulin-negative islets) | Percentage of insulin-negative islets with GPX1 staining intensity of 2 (and number of insulin-negative islets) | Percentage of insulin-negative islets with GPX1 staining intensity of 3 (and number of insulin-negative islets) | Percentage of insulin-negative islets with GPX1 staining intensity of 4 (and number of insulin-negative islets) | Percentage of insulin-negative islets with GPX1 staining intensity of 5 (and number of insulin-negative islets) | Percentage of insulin-negative islets with GPX1 staining intensity of 6 (and number of insulin-negative islets) |
| --- | --- | --- | --- | --- | --- | --- | --- |
|  |  |  |  |  |  |  |  |
| DiViD case 1, 4 weeks | 87 | 0.00% (0) | 0.00% (0) | 1.15% (1) | 4.60% (4) | 85.06% (74) | 9.20% (8) |
| DiViD case 2, 3 weeks | 27 | 0.00% (0) | 0.00% (0) | 7.40% (2) | 44.44% (12) | 46.15% (12) | 0.00% (0) |
| DiViD case 3, 9 weeks | 32 | 0.00% (0) | 0.00% (0) | 12.50% (4) | 68.75% (22) | 9.38% (3) | 9.38% (3) |
| DiViD case 4, 5 weeks | 66 | 0.00% (0) | 1.52% (1) | 65.16% (43) | 0.00% (0) | 0.00% (0) | 33.33% (22) |

**ESM Table 10.** Group 4 (long-term diabetic cases): Summary of number of insulin-negative islets and percentages of islets with various grades of GPX1 staining intensities

| Case number and duration of diabetes from diagnosis in years | Total number of insulin-negative islets | Percentage of insulin-negative islets with GPX1 staining intensity of 1 (and number of insulin-negative islets) | Percentage of insulin-negative islets with GPX1 staining intensity of 2 (and number of insulin-negative islets) | Percentage of insulin-negative islets with GPX1 staining intensity of 3 (and number of insulin-negative islets) | Percentage of insulin-negative islets with GPX1 staining intensity of 4 (and number of insulin-negative islets) | Percentage of insulin-negative islets with GPX1 staining intensity of 5 (and number of insulin-negative islets) | Percentage of insuin-negative islets with GPX1 staining intensity of 6 (and number of insulin-negative islets) |
| --- | --- | --- | --- | --- | --- | --- | --- |
|  |  |  |  |  |  |  |  |
| E560, 1.5 years | 34 | 0.00% (0) | 0.00% (0) | 17.65% (6) | 58.82% (20) | 11.76% (4) | 11.76% (4) |
| 6211, 4 years | 43 | 2.33% (1) | 2.33% (1) | 86.47% (37) | 0.00% (0) | 2.33% (1) | 6.98% (3) |
| 6088, 5 years | 71 | 0.00% (0) | 47.89% (34) | 50.70% (36) | 0.00% (0) | 0.00% (0) | 1.41% (1) |
| 6070, 7 years | 57 | 0.00% (0) | 8.78% (5) | 31.58% (18) | 8.78% (5) | 21.05% (12) | 29.82% (17) |
| 6245, 7 years | 108 | 14.81% (16) | 74.07% (80) | 8.33% (9) | 1.85% (2) | 0.00% (0) | 0.93% (1) |
| 6045, 8 years | 27 | 0.00% (0) | 25.93% (7) | 11.11% (3) | 0.00% (0) | 22.22% (6) | 40.74% (11) |
| 6262, 8 years | 104 | 0.00% (0) | 5.77% (6) | 80.77% (84) | 0.00% (0) | 0.96% (1) | 12.5% (13) |
| 6220, 11 years | 26 | 0.00% (0) | 7.69% (2) | 53.85% (14) | 0.00% (0) | 7.69% (2) | 30.77% (8) |
