# Supplementary material for: Distribution of glutathione peroxidase-1 immunoreactive cells in pancreatic islets from type 1 diabetic donors and non-diabetic donors with and without islet cell autoantibodies is variable and independent of disease
Source: Cell Tissue Res. 2025 Mar 10;400(3):255–71. doi: 10.1007/s00441-025-03955-5 (PMC12125085; doi:10.1007/s00441-025-03955-5)
Supplement: Supplementary file 4 — Supplementary file4 (DOCX 35 KB) [file 441_2025_3955_MOESM4_ESM.docx]

**ESM Table 11.** Group 1 (newly-diagnosed cases): Summary of percentages and number of cells positive for insulin or glucagon in 10 randomly selected islets per case, with various grades of GPX1 staining intensities. Each insulin and glucagon cell was separately graded for GPX1 intensity as either negative, weak, moderate or strong (refer to the guide in Fig. 2)

| Case number and duration of diabetes from diagnosis in weeks | Total number of beta cells in 10 islets | Percentage of beta cells with negative GPX1 staining intensity (and number of beta cells) | Percentage of beta cells with weak GPX1 staining intensity (and number of beta cells) | Percentage of beta cells with moderate GPX1 staining intensity (and number of beta cells) | Percentage of beta cells with strong GPX1 staining intensity (and number of beta cells) | Total number of glucagon cells in 10 islets | Percentage of glucagon cells with negative GPX1 staining intensity (and number of glucagon cells) | Percentage of glucagon cells with weak GPX1 staining intensity (and number of glucagon cells) | Percentage of glucagon cells with moderate GPX1 staining intensity (and number of glucagon cells) | Percentage of glucagon cells with strong GPX1 staining intensity (and number of glucagon cells) |
| --- | --- | --- | --- | --- | --- | --- | --- | --- | --- | --- |
| DiViD case 1, 4 weeks | 0 | 0 (0) | 0 (0) | 0 (0) | 0 (0) | 812 | 0 (0) | 10.34 (84) | 0.99 (8) | 88.67 (720) |
| DiViD case 2,  3 weeks | 334 | 5.09 (17) | 79.64 (266) | 15.27 (51) | 0 (0) | 968 | 0 (0) | 46.28 (448) | 44.94 (435) | 8.78 (85) |
| DiViD case 3, 9 weeks | 461 | 76.79 (354) | 22.55 (104) | 0.65 (3) | 0 (0) | 795 | 1.01 (8) | 8.93 (71) | 77.48 (616) | 12.58 (100) |
| DiViD case 4, 5 weeks | 428 | 0 (0) | 0 (0) | 98.83 (423) | 1.17 (5) | 1446 | 0 (0) | 3.67 (53) | 92.60 (1339) | 3.73 (54) |

**ESM Table 12.** Group 2 (non-diabetic autoantibody-negative cases): Summary of percentages and number of cells positive for insulin or glucagon in 10 randomly selected islets per case, with various grades of GPX1 staining intensities. Each insulin and glucagon cell was graded separately for GPX1 intensity as negative, weak, moderate, or strong (refer to the guide in Figure 2).

| Case number | Total number of beta cells in 10 islets | Percentage of beta cells with negative GPX1 staining intensity (and number of beta cells) | Percentage of beta cells with weak GPX1 staining intensity (and number of beta cells) | Percentage of beta cells with moderate GPX1 staining intensity (and number of beta cells) | Percentage of beta cells with strong GPX1 staining intensity (and number of beta cells) | Total number of glucagon cells in 10 islets | Percentage of glucagon cells with negative GPX1 staining intensity (and number of glucagon cells) | Percentage of glucagon cells with weak GPX1 staining intensity (and number of glucagon cells) | Percentage of glucagon cells with moderate GPX1 staining intensity (and number of glucagon cells) | Percentage of glucagon cells with strong GPX1 staining intensity (and number of glucagon cells) |
| --- | --- | --- | --- | --- | --- | --- | --- | --- | --- | --- |
|  |  |  |  |  |  |  |  |  |  |  |
| 6289 | 989 | 27.30 (270) | 69.76 (690) | 2.93 (29) | 0 (0) | 793 | 3.15 (25) | 86.51 (686) | 4.79 (38) | 5.54 (44) |
| 6234 | 1005 | 6.36 (64) | 50.14 (504) | 42.28 (425) | 1.19 (12) | 824 | 0.12 (1) | 36.52 (301) | 61.77 (509) | 1.57 (13) |
| 6160 | 1139 | 23.26 (265) | 67.25 (766) | 8.6 (98) | 0.87 (10) | 594 | 1.34 (8) | 50 (297) | 30.47 (181) | 18.18 (108) |
| 6178 | 1228 | 96.98 (1191) | 1.87 (23) | 0.97 (12) | 0.16 (2) | 778 | 65.80 (512) | 9.51 (74) | 8.35 (65) | 16.32 (127) |
| 6401 | 828 | 0 (0) | 21.37 (177) | 78.26 (648) | 0.36 (3) | 427 | 0 (0) | 4.91 (21) | 94.37 (403) | 0.70 (3) |
| 6055 | 1495 | 0 (0) | 87.55 (1309) | 12.44 (186) | 0 (0) | 646 | 0 (0) | 15.33 (99) | 82.97 (536) | 1.70 (11) |
| 6048 | 1239 | 48.18 (597) | 39.54 (490) | 12.26 (152) | 0 (0) | 806 | 45.78 (369) | 42.67 (92) | 11.41 (92) | 0.12 (1) |
| 6229 | 1124 | 8.54 (96) | 70.90 (797) | 18.77 (211) | 1.77 (20) | 1083 | 6.92 (75) | 62.12 (673) | 18.55 (201) | 12.37 (134) |
| 6369 | 1155 | 0.77 (9) | 17.83 (206) | 72.30 (835) | 9.09 (105) | 983 | 0 (0) | 4.06 (40) | 80.26 (789) | 15.66 (154) |
|  |  |  |  |  |  |  |  |  |  |  |

**ESM Table 13.** Group 3 (non-diabetic autoantibody-positive cases): Summary of percentages and number of cells positive for insulin or glucagon in 10 randomly selected islets per case, with various grades of GPX1 staining intensities. Each insulin and glucagon cell was graded separately for GPX1 intensity as negative, weak, moderate, or strong (refer to the guide in Figure 2).

| Case number | Total number of beta cells in 10 islets | Percentage of beta cells with negative GPX1 staining intensity (and number of beta cells) | Percentage of beta cells with weak GPX1 staining intensity (and number of beta cells) | Percentage of beta cells with moderate GPX1 staining intensity (and number of beta cells) | Percentage of beta cells with strong GPX1 staining intensity (and number of beta cells) | Total number of glucagon cells in 10 islets | Percentage of glucagon cells with negative GPX1 staining intensity (and number of glucagon cells) | Percentage of glucagon cells with weak GPX1 staining intensity (and number of glucagon cells) | Percentage of glucagon cells with moderate GPX1 staining intensity (and number of glucagon cells) | Percentage of glucagon cells with strong GPX1 staining intensity (and number of glucagon cells) |
| --- | --- | --- | --- | --- | --- | --- | --- | --- | --- | --- |
|  |  |  |  |  |  |  |  |  |  |  |
| 6424 | 983 | 9.76 (96) | 24.51 (241) | 65.20 (641) | 0.50 (5) | 839 | 0 (0) | 30.39 (255) | 67.69 (568) | 1.90 (16) |
| 6267 | 1005 | 0 (0) | 99.50 (1000) | 0 (0) | 0.50 (5) | 631 | 0 (0) | 73.21 (462) | 17.11 (108) | 9.66 (61) |
| 6301 | 1376 | 0 (0) | 8.28 (114) | 85.37 (1167) | 6.90 (95) | 1588 | 0 (0) | 9.88 (157) | 88.54 (1406) | 1.57 (25) |
| 6310 | 749 | 0 (0) | 62.21 (466) | 34.45 (258) | 3.33 (25) | 769 | 0 (0) | 0.52 (4) | 64.11 (493) | 35.37 (272) |
| 6167 | 948 | 0 (0) | 56.54 (536) | 34.17 (324) | 9.28 (88) | 861 | 0 (0) | 55.75 (480) | 28.69 (247) | 15.56 (134) |
| 6158 | 1134 | 0 (0) | 6.70 (76) | 90.48 (1026) | 2.82 (32) | 464 | 0 (0) | 0 (0) | 94.40 (438) | 5.60 (26) |
|  |  |  |  |  |  |  |  |  |  |  |

**ESM Table 14.** Group 4 (long-term diabetic cases): Summary of percentages and number of cells positive for insulin or glucagon in 10 randomly selected islets per case, showing various grades of GPX1 staining intensities. Each insulin and glucagon cell was graded separately for GPX1 intensity as negative, weak, moderate or strong (refer to the guide in Figure 2).

| Case number and duration of diabetes from diagnosis in years | Total number of beta cells in 10 islets | Percentage of beta cells with negative GPX1 staining intensity (and number of beta cells) | Percentage of beta cells with weak GPX1 staining intensity (and number of beta cells) | Percentage of beta cells with moderate GPX1 staining intensity (and number of beta cells) | Percentage of beta cells with strong GPX1 staining intensity (and number of beta cells) | Total number of glucagon cells in 10 islets | Percentage of glucagon cells with negative GPX1 staining intensity (and number of glucagon cells) | Percentage of glucagon cells with weak GPX1 staining intensity (and number of glucagon cells) | Percentage of glucagon cells with moderate GPX1 staining intensity (and number of glucagon cells) | Percentage of glucagon cells with strong GPX1 staining intensity (and number of glucagon cells) |
| --- | --- | --- | --- | --- | --- | --- | --- | --- | --- | --- |
|  |  |  |  |  |  |  |  |  |  |  |
| E560, 1.5 years | 413 | 0 (0) | 12.83 (53) | 74.58 (308) | 12.59 (52) | 864 | 0 (0) | 22.57 (195) | 33.45 (289) | 43.98 (380) |
| 6211, 4 years | 453 | 0 (0) | 50.55 (229) | 35.98 (163) | 13.47 (61) | 1878 | 0 (0) | 33.76 (634) | 63.04 (1184) | 3.19 (60) |
| 6088, 5 years | 0 | 0 (0) | 0 (0) | 0 (0) | 0 (0) | 1429 | 0 (0) | 31.56 (451) | 60.88 (870) | 7.55 (108) |
| 6070, 7 years | 428 | 0 (0) | 76.64 (328) | 20.33 (87) | 3.03 (13) | 1295 | 0 (0) | 30.11 (390) | 50.35 (652) | 19.54 (253) |
| 6245, 7 years | 121 | 0 (0) | 83.47 (101) | 16.53 (20) | 0 (0) | 1066 | 0 (0) | 50.09 (534) | 49.15 (524) | 0.75 (8) |
| 6045, 8 years | 0 | 0 (0) | 0 (0) | 0 (0) | 0 (0) | 463 | 0 (0) | 25.70 (119) | 57.88 (268) | 16.41 (76) |
| 6262, 8 years | 0 | 0 (0) | 0 (0) | 0 (0) | 0 (0) | 1139 | 0 (0) | 0.70 (8) | 78.31 (892) | 20.98 (239) |
| 6220, 11 years | 0 | 0 (0) | 0 (0) | 0 (0) | 0 (0) | 2225 | 0 (0) | 37.84 (842) | 54.07 (1203) | 8.09 (180) |
|  |  |  |  |  |  |  |  |  |  |  |

**ESM Table 15:** Percentage of total number of beta cells and glucagon cells in 10 randomly selected islets from each case with negative, weak, moderate, and strong staining intensities for GPX1

| Study groups and number of cases per group | Number of beta cells in 10 random islets per group | Percentage of beta cells with negative GPX1 staining intensity and (number of beta cells) | Percentage of beta cells with weak GPX1 staining intensity and (number of beta cells) | Percentage of beta cells with moderate GPX1 staining intensity and (number of beta cells) | Percentage of beta cells with strong GPX1 staining intensity and (number of beta cells) | Number of glucagon cells in random 10 islets per group | Percentage of glucagon cells with negative GPX1 staining intensity and (number of glucagon cells) | Percentage of glucagon cells with weak GPX1 staining intensity and (number of glucagon cells) | Percentage of glucagon cells with moderate GPX1 staining intensity and (number of glucagon cells) | Percentage of glucagon cells with strong GPX1 staining intensity and (number of glucagon cells) |
| --- | --- | --- | --- | --- | --- | --- | --- | --- | --- | --- |
|  |  |  |  |  |  |  |  |  |  |  |
| Group 1 (newly diagnosed cases), 4 | 1223 | 30.34 (371) | 30.25 (370) | 39.00 (477) | 0.41 (5) | 4021 | 0.20 (8) | 16.31 (656) | 59.64 (2398) | 23.85 (959) |
| Group 2 (non-diabetic, autoantibody-negative cases), 9 | 10202 | 24.42 (2492) | 48.64 (4962) | 25.44 (2596) | 1.49 (152) | 6682 | 14.81 (990) | 34.16 (2283) | 42.11 (2814) | 8.90 (595) |
| Group 3 (non-diabetic autoantibody-positive cases), 6 | 6195 | 1.54 (96) | 39.27 (2433) | 55.14 (3416) | 4.04 (250) | 5152 | 0 (0) | 26.35 (1358) | 63.27 (3260) | 10.36 (534) |
| Group 4 (long-term diabetic cases), 8 | 1415 | 0 (0) | 50.24 (711) | 40.85 (578) | 8.90 (126) | 10359 | 0 (0) | 30.6 (3173) | 56.78(5882) | 12.60 (1304) |
