# Supplementary material for: Distribution of glutathione peroxidase-1 immunoreactive cells in pancreatic islets from type 1 diabetic donors and non-diabetic donors with and without islet cell autoantibodies is variable and independent of disease
Source: Cell Tissue Res. 2025 Mar 10;400(3):255–71. doi: 10.1007/s00441-025-03955-5 (PMC12125085; doi:10.1007/s00441-025-03955-5)
Supplement: Supplementary file 5 — Supplementary file5 (PDF 1.90 MB) [file 441_2025_3955_MOESM5_ESM.pdf]

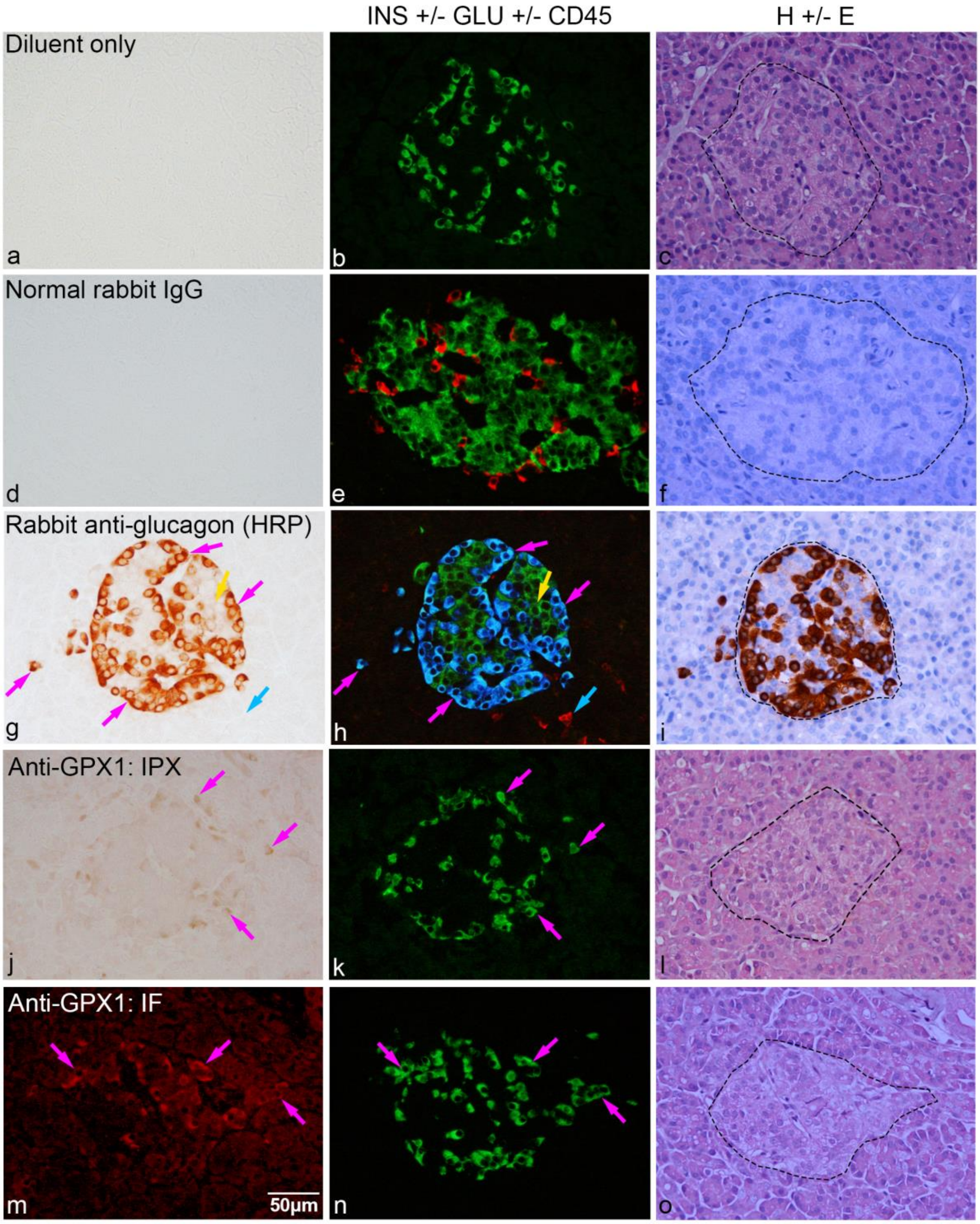


**ESM Fig. 1** Immunohistochemical specificity of rabbit anti-GPX1 from Abcam assessed on sections from non-diabetic cases from nPOD. (a) Addition of primary antibody diluent alone or normal rabbit IgG (d) to two separate sections followed by incubation with anti-rabbit IgG-horse radish peroxidase polymer and reaction with DAB show absence of staining in islets. (g,

h) Addition of rabbit anti-glucagon, guinea pig anti-insulin and mouse anti-CD45 and incubation with anti-rabbit IgG-horse radish peroxidase and DAB reaction, donkey anti-guinea pig IgG-Alexa 488 and donkey anti-mouse IgG-Alexa 568 show the expected staining of glucagon cells, beta cells and C45 cells, respectively. (j) Addition of anti-GPX1 (1:150) to a separate section followed by incubation with anti-rabbit IgG-horse radish peroxidase polymer and reaction with DAB show several GPX1 positive cells. (m) Addition of anti-GPX1 (1:150) followed by incubation with anti-rabbit IgG-Alexa 568 also shows several GPX1 positive cells. (b, k, n) Corresponding islets stained for glucagon (mouse anti-glucagon followed by addition of anti-mouse IgG Dylight 488). (e) Merged image of the corresponding islet dual-stained for insulin (green) with anti-guinea pig IgG-Alexa 488 and for glucagon (red) with donkey anti-mouse IgG-Alexa 568. (h) Merged image of the corresponding islet triple stained for insulin (green), glucagon (following conversion of brown cells to cyan) and CD45 cells (red). The third panel shows the corresponding sections counterstained with haematoxylin and eosin (c, l, o) and haematoxylin alone (f, i), where islet boundaries are indicated by black dashes. In (j, k, m, n) magenta arrows indicate some GPX1 cells positive for glucagon. (g, h) Magenta arrows point to glucagon cells, yellow arrow to insulin cells and blue arrow to CD45 cells. Scale bar in (m) 50 μm, applies to all micrographs. Anti-GPX1, antibody to glutathione peroxidase-1; E, eosin; GLU, glucagon; H, haematoxylin; IF, immunofluorescence; INS, insulin; IPX, immunoperoxidase

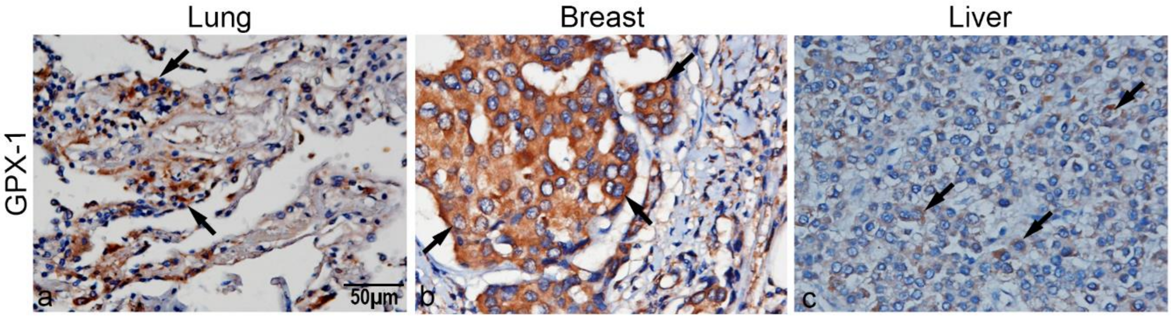


**ESM Fig. 2** Immunohistochemical staining of GPX1 in non-pancreatic tissue sections counterstained with haematoxylin. (a) lung cancer, (b) breast cancer, (c) liver cancer. Arrows indicate GPX1-positive cells in the 3 micrographs. Scale bar in (a), 50 μm, applies to all micrographs.

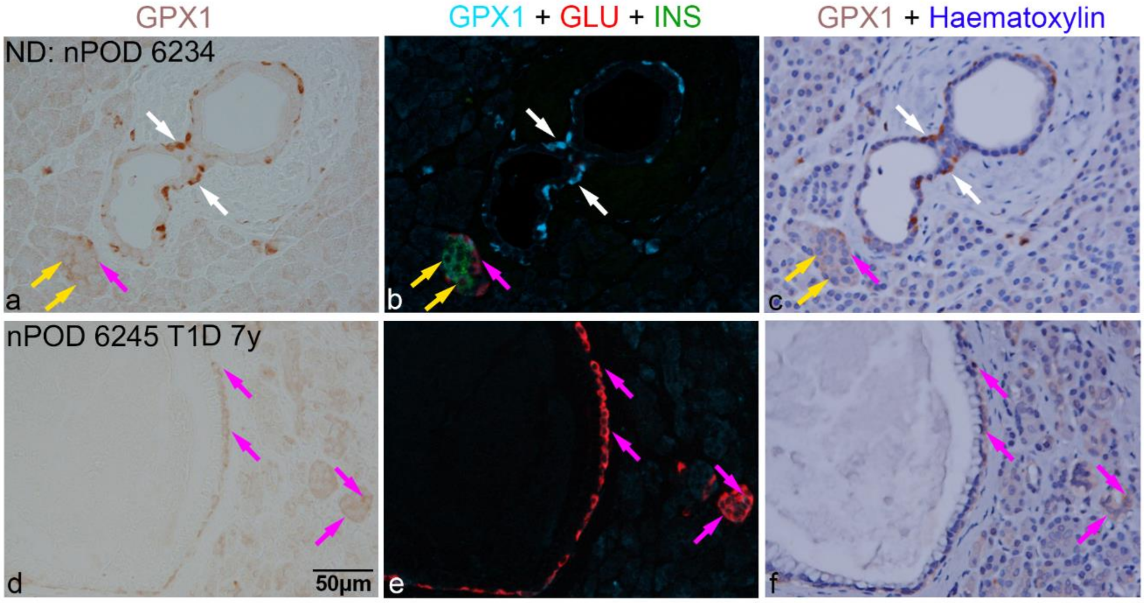


**ESM Fig. 3** Immunohistochemical staining of GPX1, insulin (green) and glucagon (red) in 2 nPOD samples showing GPX1 immunoreactivity in ductal cells (a, d). In (b) and (e) brown GPX1 positive cells were converted to blue. White arrows indicate GPX1 staining in selective ductal cells, while yellow and magenta arrows point to insulin and glucagon cells, respectively, with weak GPX1 staining. (d, e, f) Magenta arrows point to weak GPX1 staining in ductal cells positive for glucagon and in a small islet to the right of the duct (magenta arrows indicate glucagon cells (d, e, f). (c, f) Corresponding sections counterstained with haematoxylin. Scale bar in (d), 50 μm, applies to all micrographs. GLU, glucagon; GPX1, glutathione peroxidase-1; INS, insulin; T1D, type 1 diabetes; 7y, 7 years

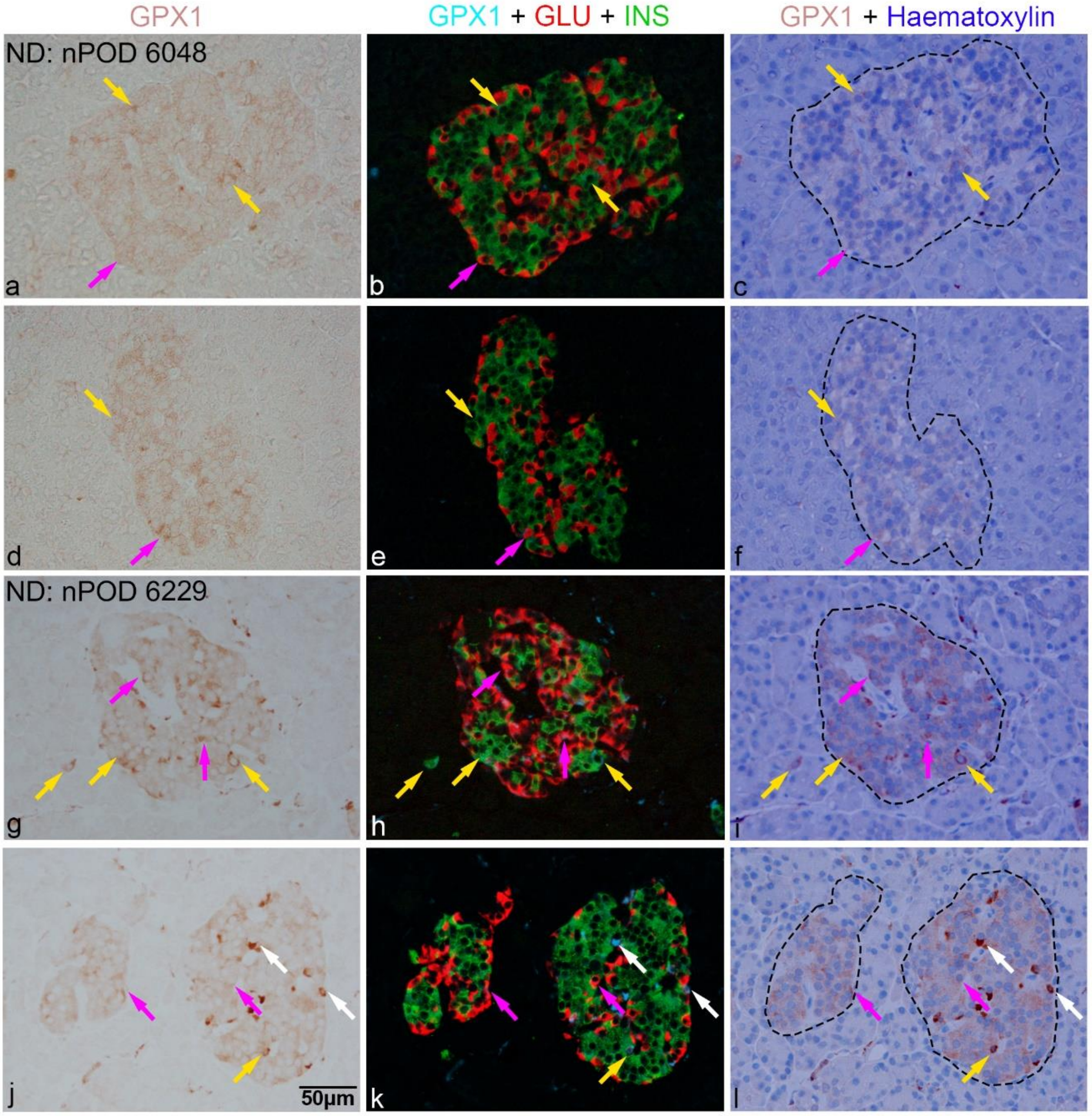


**ESM Fig. 4** Immunohistochemical analysis of pancreas sections from non-diabetic autoantibody-negative nPOD donors 6048 (a-f) and 6229 (g-l). Left panel shows GPX1-positive cells (brown) in islets or occasionally outside the islet. The middle panel shows corresponding GPX1-positive cells in blue, merged with beta (green) and alpha (red) cells, following co-staining. The third panel shows corresponding fields after counterstaining with haematoxylin, where islet boundaries are indicated by black dashes. Magenta arrows indicate GPX1 in alpha cells and yellow arrows in beta cells, while white arrows in cells negative for insulin and glucagon. Scale bar in (j), 50 µm, applies to all micrographs. GLU, glucagon; GPX1, glutathione peroxidase-1; INS, insulin; nPOD, Network for Pancreatic Organ Donors with Diabetes

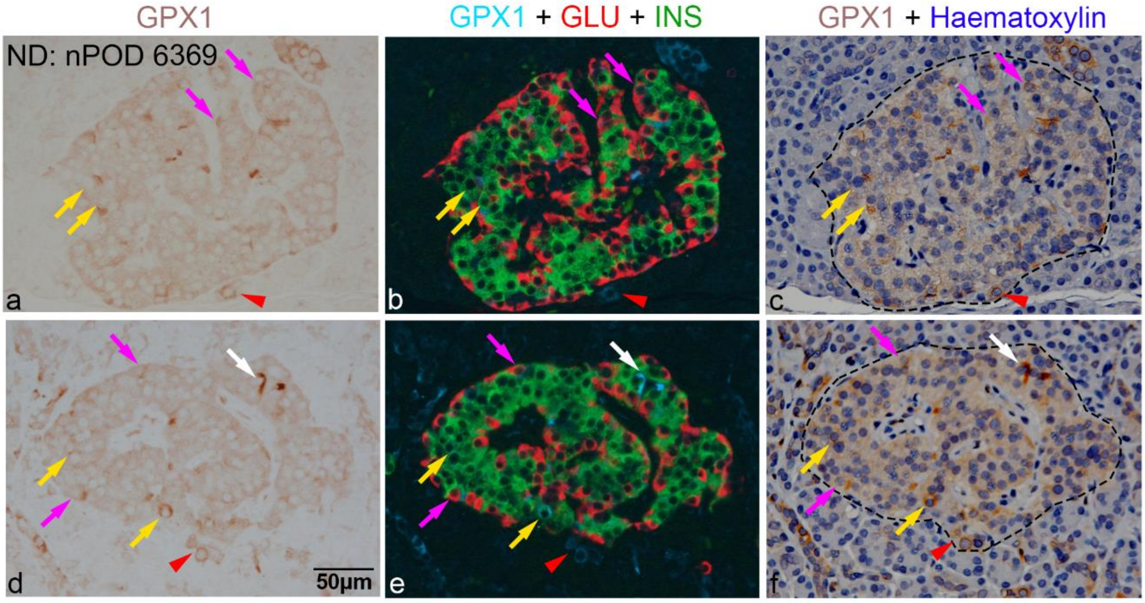


**ESM Fig. 5** Immunohistochemical analysis of pancreas sections from non-diabetic autoantibody-negative nPOD donor 6369 (a-f). Left panel shows GPX1-positive cells (brown) in islets or occasionally outside the islet. The middle panel shows corresponding GPX1-positive cells in blue, merged with beta (green) and alpha (red) cells, following co-staining. The third panel shows corresponding fields after counterstaining with haematoxylin, where islet boundaries are indicated by black dashes. Magenta arrows indicate GPX1 in alpha cells and yellow arrows in beta cells, while white arrows in cells negative for insulin and glucagon; red arrowheads indicate GPX1 staining in some insulin- and glucagon-negative cells in the exocrine region. Scale bar in (d), 50 µm, applies to all micrographs. GLU, glucagon; GPX1, glutathione peroxidase-1; INS, insulin; nPOD, Network for Pancreatic Organ Donors with Diabetes

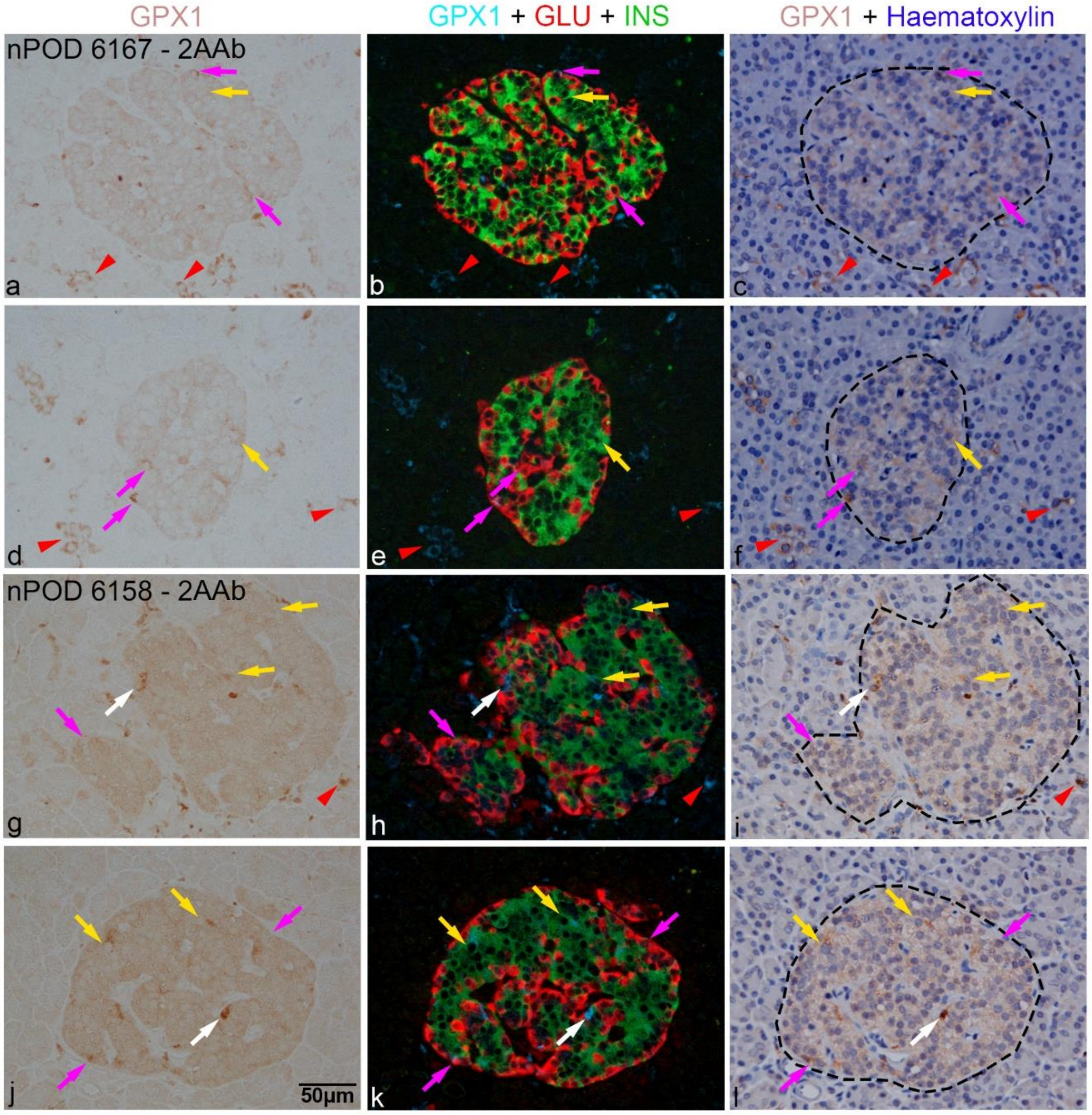


**ESM Fig. 6** Immunohistochemical analysis of pancreas sections from non-diabetic autoantibody-positive nPOD donors 6167 (a-f) and 6158 (g-l). Left panel shows GPX1-positive cells (brown) in islets or occasionally outside the islet. The middle panel shows corresponding GPX1-positive cells in blue, merged with beta (green) and alpha (red) cells, following co-staining. The third panel shows corresponding fields after counterstaining with haematoxylin, where islet boundaries are indicated by black dashes. Magenta arrows indicate GPX1 in alpha cells and yellow arrows in beta cells, while white arrows in cells negative for insulin and glucagon; red arrowheads indicate GPX1 staining in some insulin- and glucagon-negative cells in the exocrine region. Scale bar in (j), 50 µm, applies to all micrographs. AAb, autoantibodies; GLU, glucagon; GPX1, glutathione peroxidase-1; INS, insulin; nPOD, Network for Pancreatic Organ Donors with Diabetes

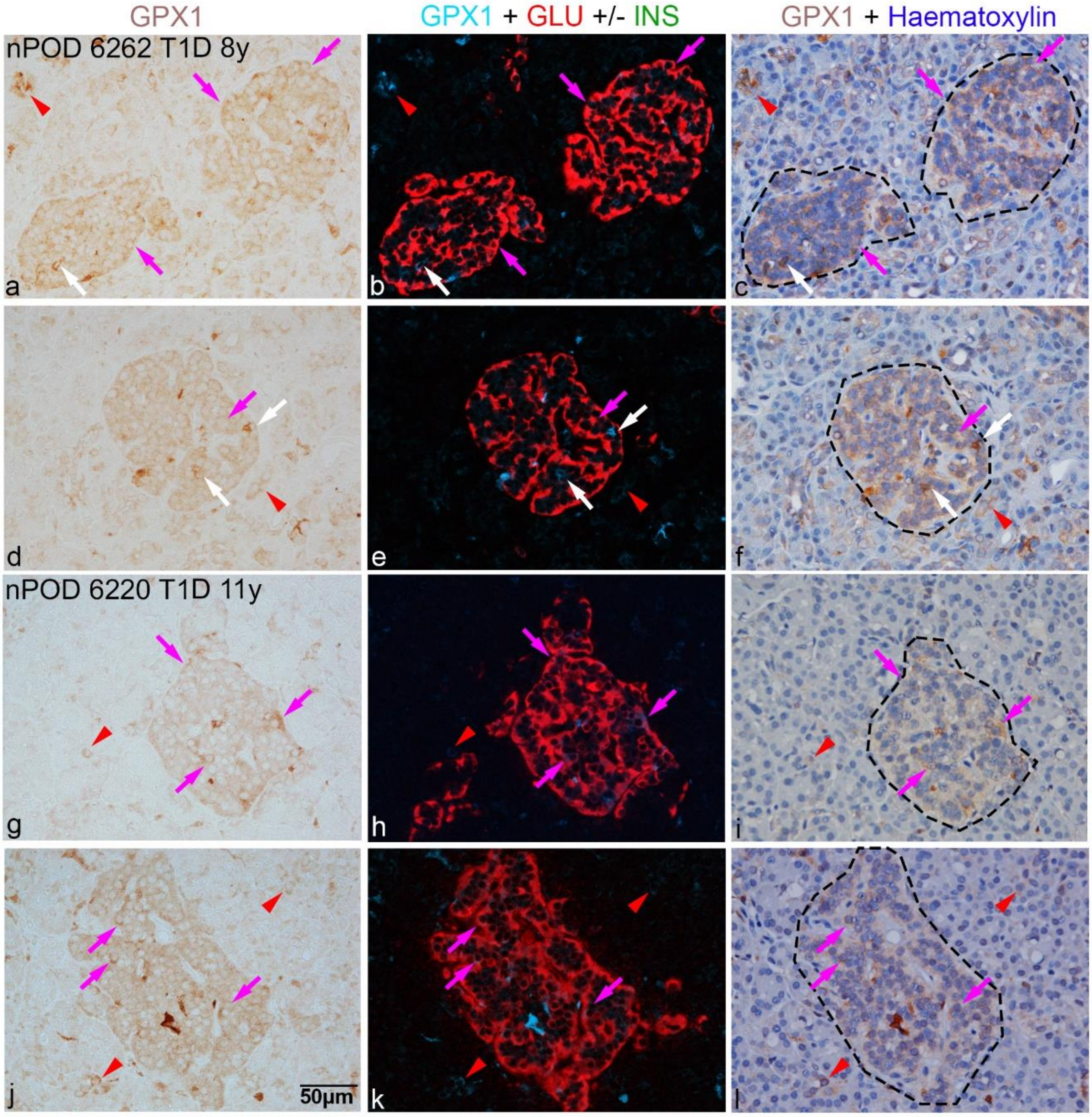


**ESM Fig. 7** Immunohistochemical analysis of pancreas sections from long-term diabetic donors nPOD 6262 (a-f; diabetes duration: 8 years) and nPOD 6220 (g-l; diabetes duration: 11 years). Left panel shows GPX1-positive cells (brown) in islets or occasionally outside the islet. The middle panel shows the corresponding GPX1-positive cells in blue, merged with alpha cells (red), following co-staining. The third panel shows corresponding fields after counterstaining with haematoxylin, where islet boundaries are indicated by black dashes. Magenta arrows indicate GPX1 in alpha cells while white arrows in cells negative for glucagon; red arrowheads indicate GPX1 staining in some insulin- and glucagon-negative cells in the exocrine region. Duration of diabetes in years is indicated in (a) and (g). Scale bar in (j), 50 μm, applies to all micrographs. GLU, glucagon; GPX1, glutathione peroxidase-1; INS, insulin; nPOD, Network for Pancreatic Organ Donors with Diabetes; T1D, type 1 diabetes
